# A Novel Approach for Drug-Target Interactions Prediction Based on Multimodal Deep Autoencoder

**DOI:** 10.3389/fphar.2019.01592

**Published:** 2020-01-28

**Authors:** Huiqing Wang, Jingjing Wang, Chunlin Dong, Yuanyuan Lian, Dan Liu, Zhiliang Yan

**Affiliations:** ^1^ College of Information and Computer, Taiyuan University of Technology, Taiyuan, China; ^2^ Dryland Agriculture Research Center, Shanxi Academy of Agricultural Sciences, Taiyuan, China

**Keywords:** drug-target interactions, multiple similarity measures, random walk with restart, positive pointwise mutual information, multimodal deep autoencoder

## Abstract

Drug targets are biomacromolecules or biomolecular structures that bind to specific drugs and produce therapeutic effects. Therefore, the prediction of drug-target interactions (DTIs) is important for disease therapy. Incorporating multiple similarity measures for drugs and targets is of essence for improving the accuracy of prediction of DTIs. However, existing studies with multiple similarity measures ignored the global structure information of similarity measures, and required manual extraction features of drug-target pairs, ignoring the non-linear relationship among features. In this paper, we proposed a novel approach MDADTI for DTIs prediction based on MDA. MDADTI applied random walk with restart method and positive pointwise mutual information to calculate the topological similarity matrices of drugs and targets, capturing the global structure information of similarity measures. Then, MDADTI applied multimodal deep autoencoder to fuse multiple topological similarity matrices of drugs and targets, automatically learned the low-dimensional features of drugs and targets, and applied deep neural network to predict DTIs. The results of 5-repeats of 10-fold cross-validation under three different cross-validation settings indicated that MDADTI is superior to the other four baseline methods. In addition, we validated the predictions of the MDADTI in six drug-target interactions reference databases, and the results showed that MDADTI can effectively identify unknown DTIs.

## Introduction

Drug targets are a kind of biological macromolecule in the body that have a pharmacodynamics function by interacting with drugs, such as certain proteins and nucleic acids. Drugs achieve disease treatment by binding specific targets and changing gene function of their targets. The prediction of drug-target interactions (DTIs) is a crucial process in drug discovery and it can facilitate the understanding of drug action mechanism, disease pathology, and drug side effect (Keiser et al., 2009; Lounkine et al., 2012; Núñez et al., 2012). Drug targets are the main carriers of drug action in drug therapy; thus, the prediction of DTIs is of great significance for disease therapy.

Drug-target interactions prediction can be viewed as a binary classification problem, where the goal is to learn a classifier that can distinguish true and false DTIs. For this problem, drug-drug similarities and target-target similarities are helpful, assuming that similar drugs tend to share similar targets and vice versa (Klabunde, 2007). Many studies applied a single similarity measure of drugs and that of targets, i.e., chemical structural similarity of drugs and amino acid sequence similarity of targets, to predict DTIs (Jacob and Vert, 2008; Yamanishi et al., 2008; Bleakley and Yamanishi, 2009; Xia et al., 2010; van Laarhoven et al., 2011; Gönen, 2012). However, both drugs and targets have different types of similarity measures and they utilize different attributes of drugs and targets, such as gene expression similarities of drugs and target proteins, drug side-effect-based similarity, proximity in protein-protein interactions and so on. It is demonstrated that drugs with similar expression patterns are likely to share common target proteins (Hizukuri et al., 2015; Vilar and Hripcsak, 2016) and drugs with similar target protein binding profiles tend to cause similar side effects, implying a direct correlation between target protein binding and side-effect similarity (Campillos et al., 2008; Hizukuri et al., 2015). Thus, only utilizing chemical structural similarity of drugs and amino acid sequence similarity of targets may miss information that is relevant to predicting new interactions.

With the development of high-throughput sequencing technology, massive multi-omics data have been generated, which provide abundant resources for predicting DTIs, including drug-side-effect association data from SIDER2 (Kuhn et al., 2015), drug-disease association data and target protein-disease association data from KEGG Disease (Kanehisa et al., 2016), protein-protein interaction data from HIPPIE (Alanis-Lobato et al., 2016), etc. Based on these data, a variety of similarity measures for drugs and targets can be calculated, which describe characteristics of drugs and targets from various aspects, and there is information complementarity among them. Thus, methods for predicting DTIs using multiple similarity measures of drugs and multiple similarity measures of targets are generated.

Perlman et al. used forward selection and backward elimination for feature selection. They selected 10 features from 15 features consisting of 5 similarity measures of drugs and 3 similarity measures of targets, and they applied logistic regression classifier to predict DTIs (Perlman et al., 2011). Olayan et al. used multiple similarity networks of drugs and multiple similarity networks of targets to construct a heterogeneous network with the known drug-target interaction network, and then they manually extracted 12 different path-category-based features from it; finally, they applied random forest to predict DTIs (Olayan et al., 2017). Nascimento et al. linearly weighted 10 drug similarity measures and 10 target similarity measures to obtain the feature of drugs and targets, respectively, and then they computed the Kronecker product of them as the feature of drug-target pairs that were fed into Kronecker regularized least squares (KronRLS) to predict DTIs (Nascimento et al., 2016). Hao et al. used Similarity Network Fusion (SNF) method to fuse two similarity measures of drugs and two similarity measures of targets into one drug similarity measure and one target similarity measure, respectively, forming features of drugs and targets, and then input them into dual network integrated logistic matrix factorization (DNILMF) to predict DTIs (Hao et al., 2017). Zheng et al. linearly weighted two similarity measures of drugs and three similarity measures of targets as the feature of drugs and targets, respectively, and then they applied Multiple Similarities Collaborative Matrix Factorization (MSCMF) to predict DTIs (Zheng et al., 2013). Compared with methods using a single similarity measure of drugs and targets, these methods achieved more accurate predictions because of fusing multiple similarity measures.

The similarity measure of drugs (targets) can be regarded as a similarity network with drugs as nodes and drug-drug similarity values as the weights of edges. These methods mentioned above directly applied multiple similarity measures to predict DTIs that only calculated the similarity between two nodes in isolation and did not consider the global topological connectivity patterns within network, ignoring the global structure information of the similarity network. Researches demonstrated that considering the global structure of network can improve the performance (Köhler et al., 2008; Fang and Gough, 2013; Peng et al., 2018). In addition, these methods relied on manual extraction features of drug-target pairs, ignoring the non-linear relationship among features, and failed to provide satisfactory prediction results.

Deep learning is a deep neural network structure with multiple hidden layers. It combines low-level features to form more abstract high-level features, discovering effective feature representations of data. Compared with traditional machine learning methods, the greatest advantage of deep learning methods is that they can extract features automatically, which do not need to perform data processing, such as feature selection, dimension reduction, format conversion, and so on. A number of studies applied deep learning to learn high-level features from the training data automatically and predict bioinformatics tasks (Pan et al., 2016; Deng et al., 2017; Fu and Peng, 2017; Gligorijević et al., 2018). Fu et al. used stacked autoencoder to learn high-level features from miRNA and disease similarity automatically, and then these features were passed to Deep Neural Network (DNN) to predict miRNA-disease associations (Fu and Peng, 2017). Pan et al. extracted raw sequence composition features from RNA and protein sequences, then applied stacked autoencoder to learn hidden high-level features, which are fed into random forest to predict RNA-protein interactions (Pan et al., 2016). These studies demonstrated that deep learning has powerful ability to learn high-level features from original data automatically, which greatly enhanced the performance of the methods and made them show satisfactory results. Gligorijević et al. proposed a new deep learning model-Multimodal Deep Autoencoder (MDA). They applied MDA to learn low-dimensional features of proteins from multiple networks and realized the fusion of multiple networks. Finally, they trained SVM with low-dimensional features of proteins to predict protein functions and achieved great performance (Gligorijević et al., 2018).

Therefore, to automatically learn features from multiple similarity measures to predict DTIs, we introduced MDA and proposed MDADTI, a novel approach for drug-target interactions prediction based on MDA. MDADTI applied Random Walk with Restart (RWR) method and Positive Pointwise Mutual Information (PPMI) to calculate topological similarity matrices of drugs (targets), capturing the global structure information of similarity measures. Then it fused multiple topological similarity matrices of drugs and targets with MDA to automatically learn the low-dimensional features of drugs and targets. Finally, it sent them to Deep Neural Network (DNN) for predicting DTIs. Furthermore, we validated the predictions of the MDADTI in drug-target interactions reference databases.

## Materials and Methods

**Figure 1:**
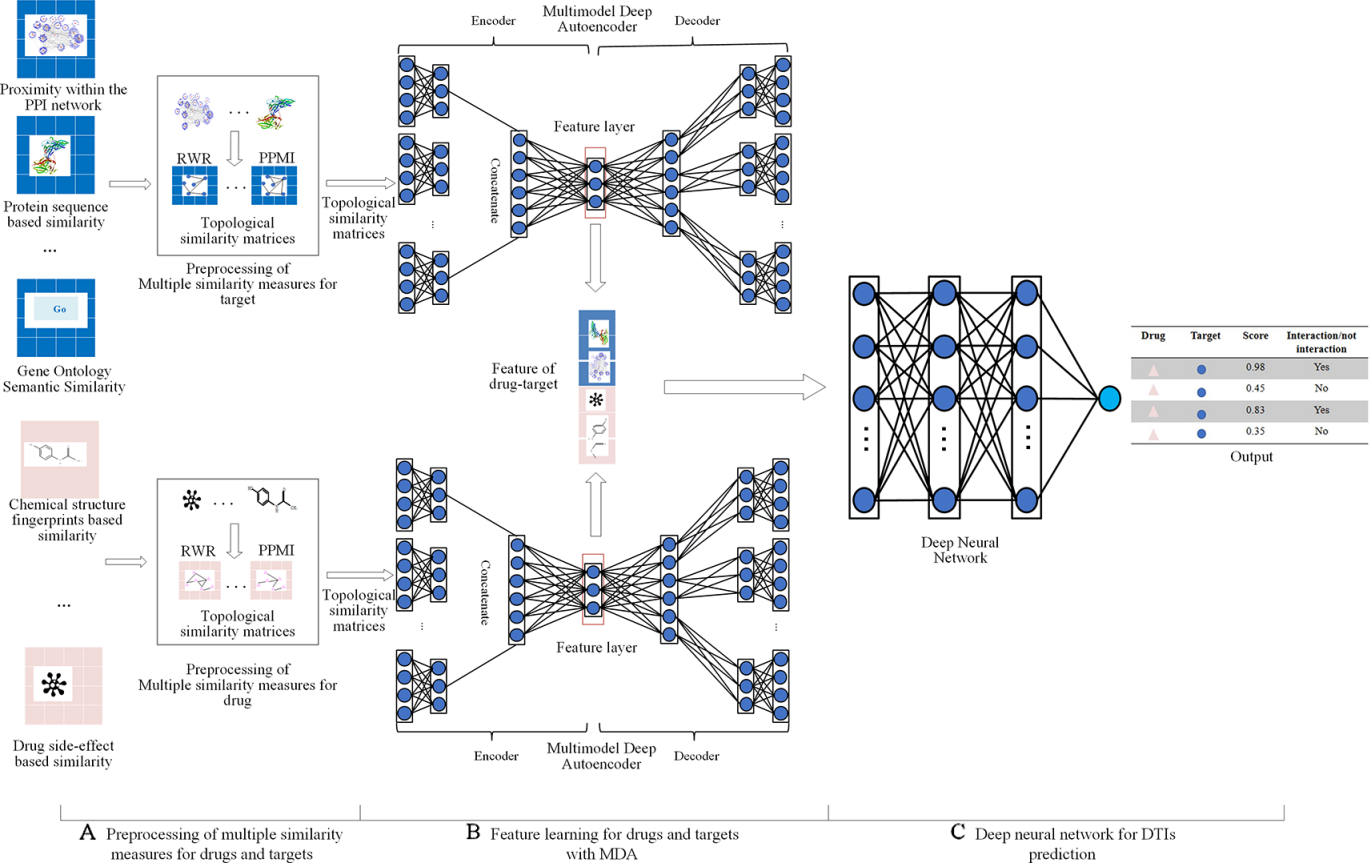
The overall framework of MDADTI method. **(A)** MDADTI applied RWR method and PPMI to calculate topological similarity matrices of drugs (targets); **(B)** MDA was applied to fuse multiple topological similarity matrices of drugs (targets) and automatically learned the low-dimensional features of drugs (targets); **(C)** DNN was applied to predict DTIs.

Multiple similarity measures of drugs (targets) describe the drug-drug similarity from various aspects, such as drug side-effects and chemical structure. Multiple similarity measures can provide complementary information for drugs or targets. Combining multiple similarity measures can improve prediction accuracy. Existing methods for predicting DTIs with multiple similarity measures directly took multiple similarity measures as inputs, ignoring their global structure information. Moreover, they required manual extraction features of drug-target pairs, limiting the size of the dataset used to train the model, ignoring the non-linear relationship among features, resulting in the lower predictive performance. Multimodal Deep Autoencoder (MDA) can fuse multiple similarities and learn high-level features automatically. This paper proposed a novel approach MDADTI based on MDA to predict drug-target interactions. MDADTI first applied Random Walk with Restart (RWR) method and Positive Pointwise Mutual Information (PPMI) to calculate topological similarity matrices of drugs (targets), capturing global structural information of each similarity measure; then it fused multiple topological similarity matrices of drugs (targets) with MDA, and realized the automatic learning and dimension reduction of drug features (target features); finally, the extracted low-dimensional features were sent into Deep Neural Network (DNN) to predict DTIs. **Figure 1** shows the overall framework of the MDADTI method.

### Materials

We evaluated the performance of our method with five datasets, including enzyme (E), ion channels (IC), G-protein-coupled receptors (GPCR), nuclear receptors (NR), and DrugBank_FDA. Each dataset contains 3 types of data: (1) DTIs data; (2) multiple similarity measures for drugs; (3) multiple similarity measures for targets.

These five datasets (E, NR, IC, GPCR, and DrugBank_FDA) were provided by Olayan et al., 2017. The DTIs data of E, NR, IC, and GPCR were originally collected by Yamanishi et al., 2008 and have been applied to many drug-target interactions prediction studies (Mei et al., 2012; Ba-Alawi et al., 2016; Lim et al., 2016; Lu et al., 2017). The multiple similarity measures for drugs and targets we used in this paper in the four datasets were computed by Nascimento et al., 2016 in the first place. DrugBank_FDA dataset was extracted from 5.0.3 version of DrugBank database (Wishart et al., 2007). It only included DTIs information of drugs approved by the FDA and single human target proteins; these proteins are not part of protein complexes. Multiple similarity measures of DrugBank_FDA for drugs and targets were computed by Olayan et al., 2017.

**Table 1** is the summary of drug-target interactions data in five datasets. As can be seen from **Table 1**, the number of negative interactions is larger than that of positive interactions in these five datasets called imbalanced data, which can reduce the predictive performance. Thus, we applied Synthetic Minority Over-sampling Technique (SMOTE) (Chawla et al., 2002) to manage the imbalanced datasets. SMOTE can syntactically generate positive samples of these datasets to balance the minority class and enhance the prediction efficiency of the classifier (Waris et al., 2016; Khan et al., 2017).

**Table 1:** Summary of drug-target interaction data.

Datasets	Number of drugs	Number of targets	Number of positive interactions	Number of negative interactions	Total number of interactions
NR	54	26	90	1314	1404
GPRC	223	95	635	20550	21185
IC	210	204	1476	41364	42840
E	445	664	2926	292554	295480
DrugBank_FDA	1482	1408	9881	2076775	2086656

**Table 2** shows a variety of similarity measures for drugs and targets in five datasets used in this paper. In NR, GPCR, IC, and E datasets, for drugs, the similarities of drugs were calculated based on distinct chemical structure fingerprints, side-effects profiles; nine various similarity measures of drugs were obtained. For targets, various amino acid sequence profiles of proteins, different parameterizations of the Mismatch (MIS) and the Spectrum (SPEC) kernels, and target proteins functional annotation based on Gene Ontology (GO) terms, proximity in the protein-protein interaction (PPI) network were considered as target information source to measure and calculate the similarities of targets; nine various similarity measures of targets were obtained.

**Table 2:** Summary of multiple similarity measures of drugs and targets.

Dataset	Entity	Information source	Similarity measures
	Drug	Chemical structure fingerprints	TAN-Tanimoto Kernel LAMBDA-Lambda-k Kernel MARG-Marginalized Kernel MINMAX-MinMax Kernel SIMCOMP-Graph kernel SPEC-Spectrum Kernel
		Side-effects	AERS-bit-AERS bit AERS-freq-AERS freq SIDER-Side-effects Similarity
	Target	Functional annotation	GO - Gene Ontology Semantic Similarity
		Sequences	MIS-k3m1-Mismatch kernel (k = 3, m = 1) MIS-k4m1-Mismatch kernel (k = 4, m = 1) MIS-k3m2-Mismatch kernel (k = 3, m = 2) MIS-k3m2-Mismatch kernel (k = 4, m = 2) SPEC-k3-Spectrum kernel (k = 3) SPEC-k4-Spectrum kernel (k = 4) SW-Smith-Waterman alignment score
		Protein-protein Interactions	PPI-Proximity in protein-protein network
DrugBank_FDA	Drug	Molecular fingerprints	CDK_Standard, CDK_Graph, CDK_Extended, CDK_Hybridization, KR, MACCS, PubChem, SIMCOMP, EC4, FC4, EC6, FC6, Lambda, Marginalized, MinMaxTanimoto, Tanimoto, Spectrum
		ATC code	_FDA_FirstLevel, FDA
		Drug interaction profile	D_interactions_FDA
		side-effects	SIDER-Side-effects Similarity
		Drug- induced gene expression	Cmap_v2_MCF7
		Drug pathways profiles	KEGG_Drug_2_Pathway
		Drug disease profiles	KEGG_Drug_Compound_ DGroup_2_Disease
	Target	Amino acid sequence	mismatch_kernel_3_1, mismatch_kernel_3_2, mismatch_kernel_4_1, mismatch_kernel_4_2, spectrum_kernel_3, spectrum_kernel_4 Merged_SWAlign_Edited
		GO annotations	CC_WANG_BMA _GO_similarity, BP_Wang_BMA_combined, MF_Wang_BMA_combined
		Proximity in the PPI network	shortest_path_networkX_distance_UP_ID_Sim_Perlman, shortest_path_networkX_ distance_UP_ID_Sim_Dnorm
		Protein domain profiles	protein2ipr_binaryMatrix _cosSim, protein2ipr_binaryMatrix _jaccardSim
		Gene expression similarity profiles	Cmap_v2_MCF7
		Target disease profiles	KEGG_Gene_2_Disease
		Target pathway profiles	KEGG_Gene_2_Pathway

In DrugBank_FDA dataset, different similarity measures between drugs were computed based on the following: different types of molecular fingerprints, drug interaction profile, drug side-effects profile, drug profile of the anatomical therapeutic class (ATC) coding system, drug-induced gene expression profile, drug-disease profiles, and drug pathways profiles; 25 various similarity measures of drugs were obtained. Furthermore, different similarity measures of target proteins were calculated based on the following protein amino acid sequence, their GO annotations, proximity in the PPI network, protein domain profiles and gene expression similarity profiles of protein encoding genes; 17 various similarity measures of targets were obtained. Chemical structures of drugs were extracted from DrugBank (Wishart et al., 2007), while the target protein sequences were extracted from UniProt (Boutet et al., 2016).

### Methods

#### Problem Description

We defined a set of DTIs and it is composed of a set of drugs D = { *d*_*i*_,*i* = 1,......, *n*_*d*_ } and a set of targets T = { *t*_*j*_,*j* = 1,......,*n*_*t*_ }, where *n*_*d*_ represents the number of drugs and *n*_*t*_ represents the number of targets. We also defined the interactions between D and T as a binary matrix Y whose element values are 0 or 1, where *y*_*ij*_ = 1 represents the drug *d*_*i*_ interacts with the target *t*_*j*_. We defined the set of similarity matrices between drugs in D as $\hat{D_{S}}$, whose dimensions are *n*_d_*n_*d*_; Similarly, we also defined the set of similarity matrices between targets in T as $\hat{T_{S}}$, whose dimensions are *n*_t_*n_*t*_. Element values in different similarity matrices represent how much drugs or targets are similar to each other based on different measures. The values of all elements in each matrix are in the range of [0, 1]. Our goal is to predict novel (i.e., unknown) interactions in Y based on the matrix Y, similarity matrices of drugs in $\hat{D_{S}}$ and similarity matrices of targets in $\hat{T_{S}}$.

### Preprocessing of Multiple Similarity Measures

A similarity matrix of drugs can be regarded as a similarity network with drugs as nodes and drug-drug similarity values as the weights of edges. The similarity network of drugs only calculates the similarity between two drug nodes in isolation and does not consider the relation among more drugs, thus cannot directly include the global structure information of the network. The topological similarity of drugs can describe the topological similarity between all pair of drug nodes in the similarity network. If the topological similarity value between two drug nodes is much larger, it indicates that they have similar positions in the similarity network and have similar functions. The topological similarity of drugs includes both the original information of the similarity network and its global structure information. Therefore, the topological similarity of drugs can solve the problem of losing information caused by original similarity network, which only considers the similarity between two drugs nodes and ignores the global structure of the similarity network. In this paper, we applied Random Walk with Restart (RWR) method and Positive Pointwise Mutual Information (PPMI) (Cao et al., 2016; Fan et al., 2019) to calculate the topological similarity of drugs in each similarity network and capture the global structure information of the similarity network. The detailed process is as follows:

(1) Given a similarity network$\;\hat{D_{S}}$ = {*S* ^(1)^,.…..,*S* ^(*n*)^}, we performed RWR on each similarity network *S* ^(*j*)^ in $\hat{D_{S}}$ to obtain the topology structure feature of drug nodes. The RWR approach can be formulated as the following recurrence relation:

(1) $$p_{i}^{\left(t\right)}=\alpha p_{i}^{\left(t-1\right)}\hat{S^{\left(j\right)}}+\left(1-\alpha \right)p_{i}^{\left(0\right)}$$

where $p_{i}^{\left(t\right)}$ is a row vector of drug *i* and its *e*th element indicates the probability of reaching the *e*th drug node after *t* steps starting from drug *i*, $p_{i}^{\left(0\right)}$ is the initial one-hot vector, *α* is the probability of restart, and $\hat{S^{\left(j\right)}}$ is the one-step probability transition matrix obtained by applying row-wise normalization of the similarity matrix *S* ^(*j*)^. The calculation formula of the topological structure feature of drug node *i* is as follows:

(2) $$p_{i}=\sum_{t=1}^{T}p_{i}^{\left(t\right)}$$

where *T* is the total number of random walk steps. Repeat this process for each node *i* in the similarity network *S* ^(*j*)^ to obtain topology feature matrix$\;P^{\left(S^{\left(j\right)}\right)}\in R^{n_{d}\times n_{d}}$.

(2) Based on the topological structure feature matrix $P^{\left(S^{\left(j\right)}\right)}$, we applied PPMI (Chen et al., 2016) to calculate the topological similarity between all pair of nodes, and obtained the topological similarity matrix $X^{\left(S^{\left(j\right)}\right)}\in R^{n_{d}\times n_{d}}$ of the similarity network *S* ^(*j*)^, capturing the global structure information. The topological similarity between node *i* and node *k* is defined as:

(3) $$X_{ik}^{\left(S^{\left(j\right)}\right)}=\text{max}\left(0,\log_{2}\frac{P_{ik}^{\left(S^{\left(j\right)}\right)}\sum_{i}\sum_{k}P_{ik}^{\left(S^{\left(j\right)}\right)}}{\sum_{i}P_{ik}^{\left(S^{\left(j\right)}\right)}\sum_{k}P_{ik}^{\left(S^{\left(j\right)}\right)}}\right)$$

where $P_{ik}^{\left(S^{\left(j\right)}\right)}$represents the elements of the *i*th row and the *k*th column of the topological structure feature matrix ${\text{P}}^{\left(S^{\left(j\right)}\right)}$.

The preprocessing procedure for multiple similarity measures of targets is the same as that of drugs.

#### Feature Learning for Drugs and Targets With MDA

**Figure 2:**
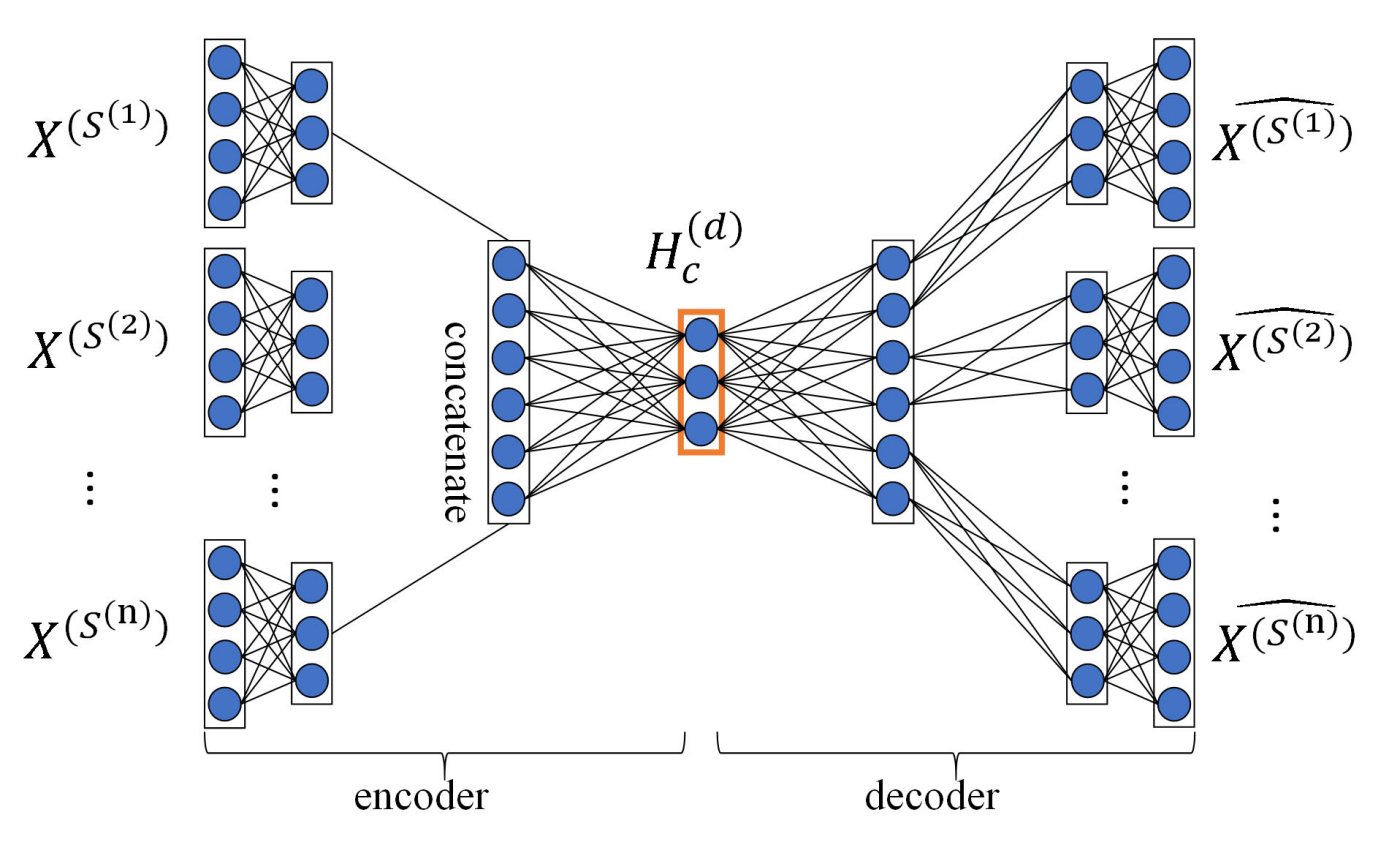
Structure diagram of MDA. The MDA consists of two parts: encoder and decoder, the inputs of encoder are multiple topological similarity matrices $X^{\left(S^{\left(j\right)}\right)}$, the hidden layer in the red box is feature layer whose output is the low-dimensional feature matrix of drugs $H_{c}^{\left(d\right)}$, the output of decoder are multiple reconstructed topological similarity matrices $\hat{X^{\left(S^{\left(j\right)}\right)}}$.

Fusing multiple drug-drug similarity measures and multiple target-target similarity measures contributes to obtaining abundant information about drugs and targets. Capturing non-linear relationships among features can improve the accuracy of DTIs prediction. Therefore, we applied MDA to fuse multiple similarity measures of drugs and targets and automatically learn low-dimensional feature matrices of drugs and targets, respectively, capturing the non-linear relationship among features. After the pretreatment, we obtained multiple topological similarity matrices of drugs $X^{\left(S^{\left(j\right)}\right)}\in R^{n_{d}\times n_{d}},j\in \left[1,\ldots \ldots n\right]$ that contain both original information of similarity measures and their global structure information. In this paper, we applied MDA to fuse multiple topological similarity matrices of drugs and automatically learn the low-dimensional feature matrix of drugs $H_{c}^{\left(d\right)}\in R^{n_{d}\times d}$. As an unsupervised neural network model, MDA uses backpropagation algorithm to train and adjust the model parameters, so that the input data can still be restored to the original features by encoding and decoding process. The structure of MDA is shown in **Figure 2**.

Encoding is the process that MDA learns the hidden features of input data with multi-layer non-linear functions. We first calculated the non-linear embedding $H^{\left(S^{\left(j\right)}\right)}$ of each topological similarity matrix $X^{\left(S^{\left(j\right)}\right)}$ in the first hidden layer of MDA:

(4) $$H^{\left(S^{\left(j\right)}\right)}=\sigma \left(W_{1}^{\left(S^{\left(j\right)}\right)}X^{\left(S^{\left(j\right)}\right)}+B_{1}^{\left(S^{\left(j\right)}\right)}\right)$$

where $W_{1}^{\left(S^{\left(j\right)}\right)}$ and $B_{1}^{\left(S^{\left(j\right)}\right)}$ are weight matrix and bias matrix, j∈[1,…n], $\sigma \left(x\right)=\frac{1}{1+e^{-x}}$ is the sigmoid activation function.

Then, we computed the low-dimensional feature matrix of drugs $H_{c}^{\left(d\right)}\in R^{n_{d}\times d}$ by applying multiple non-linear functions (i.e., multiple hidden layers) on the feature representation obtained by concatenating features from all topological similarity matrices obtained in the previous layer:

(5) $$H_{c}^{\left(d\right)}=\sigma \left(W_{1}\left[H^{\left(S^{\left(1\right)}\right)},\ldots \ldots ,H^{\left(S^{\left(n\right)}\right)}\right]+B_{1}\right)$$

where $\left[H^{\left(s^{\left(1\right)}\right)},\text{......},H^{\left(s^{\left(n\right)}\right)}\right]$is the concatenated matrix of N embedding $H^{\left(S^{\left(j\right)}\right)}$ obtained in the previous layer; *W*_1_ and *B*_1_ are weight matrix and bias matrix, and *σ*(*x*) is the sigmoid activation function.

Decoding is the process that MDA reconstructs input data from hidden features with multi-layer non-linear functions. Hidden features are obtained through encoding process. We reconstructed multiple topological similarity matrices $\hat{X^{\left(S^{\left(j\right)}\right)}}$ from the feature matrix $H_{c}^{\left(d\right)}$ of drugs with a multi-layer non-linear function:

(6) $$\hat{X^{\left(S^{\left(j\right)}\right)}}=\sigma \left(W_{2}^{\left(S^{\left(j\right)}\right)}H_{c}^{\left(d\right)}+B_{2}^{\left(S^{\left(j\right)}\right)}\right)$$

where $W_{2}^{\left(S^{\left(j\right)}\right)}$ and $B_{2}^{\left(S^{\left(j\right)}\right)}$ are weight matrix and bias matrix, j∈ [1, ……, n], *σ*(*x*) is the sigmoid activation function.

To get the feature matrix of drugs $H_{c}^{\left(d\right)}$, MDA obtained the unknown parameters θ in the encoding and decoding process by minimizing the reconstruction error of $X^{\left(S^{\left(j\right)}\right)}$ and $\hat{X^{\left(S^{\left(j\right)}\right)}}$:

(7) θ^=argminL(θ)= θargmin∑j=1  nloss(X(S(j)),X(S(j))^)

where $\theta =\left\{W_{1},B_{1},W_{1}^{\left(S^{\left(j\right)}\right)},B_{1}^{\left(S^{\left(j\right)}\right)},W_{2}^{\left(S^{\left(j\right)}\right)},B_{2}^{\left(S^{\left(j\right)}\right)}\right\}$ is the set of unknown parameters in the encoding and decoding process, and *n* represents the number of drug topological similarity matrices, and *loss*(*) is cross-entropy function.

The learning process of the feature matrix $H_{c}^{\left(t\right)}$of targets is the same as that of feature matrix $H_{c}^{\left(d\right)}$of drugs. The hyperparameters of training MDA include epoch, batch size, and learning rate with values of 100, 32, and 0.001, respectively.

#### Deep Neural Network for DTIs Prediction

We formulated the problem of DTIs prediction as a binary classification problem. We introduced Deep Neural network (DNN) to predict DTIs. The DNN of our method consists of 5 fully-connected layers, including 1 input layer, 3 hidden layers, and 1 output layer. The choice of the number of hidden layers depends on experiments. After a lot of experiments, MDADTI achieved best predicted results when DNN consists of 3 hidden layers and the number of each layer is 300, 200, and 100. All the neuron units in the layer *i* are connected to the previous layer (*i-1*) and then generated outputs with non-linear transformation function *f*:

(8) $$o_{j}=f\left(\sum_{i=1}^{H}w_{i}o_{i}+b_{i}\right)$$

where *H* is the number of neurons in hidden layer; ${\left\{w_{i,}b_{i}\right\}}_{i=1}^{H}$ are the weights and bias of neuron j which sums up all the hidden units; *f*(*) is Relu activation function, which is a non-linear function that can capture hidden patterns in the input data (Chen et al., 2016) and can reduce gradient vanishing at the same time.

In order to predict DTIs, we concatenated the feature matrix of drugs $H_{c}^{\left(d\right)}$and the feature matrix of targets $H_{c}^{\left(t\right)}$to get the feature matrix of drug-target pairs *H*_*c*_. Then we used *H*_*c*_ to train DNN, and the final output layer utilized $sigmoid=\frac{1}{1+e^{-x}}$ function to predict the interaction possibility of the drug-target pair. If the probability exceeds 0.5, we determine that there is potential interaction between the drug and the target.

#### Model Training

MDADTI was trained using the Keras 1.0.1 library with Tensorflow as the backend. The model utilized a backpropagation algorithm to calculate the loss function value between the output and the label, then it calculated its gradient relative to each neuron, and updated the weight according to the gradient direction. We chose cross-entropy function as the loss function:

(9) $$C=-\frac{1}{n}\sum_{x}\sum_{t}\left[y\ln\alpha +\left(1-y\right)\ln\left(1-\alpha \right)\right]$$

where *C* is the output of cross-entropy cost function, *x* represents the index of the training samples (i.e., drug-target pairs), *t* represents the index of different labels, *y* represents the true label for sample *x* whose value is 0 or 1, and *a* represents the predicted output for sample *x*. Since the closer the predicted output is to the true label, the smaller C value we can get, our goal is to minimize the cross-entropy function to get the best prediction of DTIs.

In the process of training the model, choosing a good optimizer not only accelerate the training of the model but also contribute to obtaining relatively good experimental results. It is observed that momentum-based stochastic gradient descent (SGD) can effectively train deep learning models (Sutskever et al., 2013). Thus, we chose SGD with momentum as optimizer to minimize the objective function.

Overfitting is a common problem in deep learning. It means that the model works well on the training set, and its predictive effect on the test set is poor, which results in weak generalization ability of the model. We used Dropout and EarlyStopping to prevent overfitting. Dropout (Srivastava et al., 2014) is a common regularization technique in neural networks, referring to randomly ‘dropping' (i.e., setting to zero) the output of a neuron with some fixed probability *p*. It means that the start-up effects on the downstream of these neurons are neglected in the forward propagation, and the weights are not updated in the backpropagation. The effect of dropout is that the network is less sensitive to the change of the weight of a certain neuron; it also leads to increased generalization ability and reduced overfitting. We used dropout in each fully connected layer of DNN and set dropout rate of *p* = 0.5, which seems to be close to optimal for a wide range of networks and tasks (Srivastava et al., 2014). EarlyStopping refers to stopping training model when the performance of the model on the validation set begins to decline. Thus, the overfitting problem caused by overtraining can be avoided. We implemented EarlyStopping by training our model with the training set and computing the accuracy on the validation set. We monitored the accuracy of MDADTI on validation set at the end of every epoch and stop the training when accuracy does not rise for 10 consecutive epochs.

## Results

### Experimental Setup and Model Evaluation

In this paper, we applied the area under the ROC (receiver-operating characteristics) curve (AUC) and the area under the precision-recall curve (AUPR) to evaluate the performance of MDADTI model. An AUC value of 1 indicates that the performance is perfect, and an AUC value of 0.5 indicates random predictive performance. Similar to the AUC score, AUPR values closer to 1 indicates that the performance is better. The calculation formulas for True Positive Rate (TPR), False Positive Rate (FPR), and precision and recall related to AUC and AUPR are as follows:

(10) TPR=recall=TP/(TP+FN)

(11) FPR=FP/(FP+TN)

(12) precision=TP/(TP+FP)

where TP represents true positive, TN represents true negative, FP represents false positive, and FN represents false negative; these formulas are based on the confusion matrix.

The performance of DTIs prediction methods was evaluated under 5-repeats of 10-fold cross-validation (CV), and both AUC and AUPR were used as the evaluation metrics. We calculated an AUC score in each repetition of CV and reported a final AUC score that was the average over the five repetitions. The AUPR score was calculated in the same manner. The drug-target interaction matrix Y has *n*_*d*_ rows for drugs and *n*_*t*_ columns for targets. We conducted CV under three different settings as follows:

- CVS1: CV on drug-target pairs—random entries in Y (i.e., drug-target pairs) were selected for testing.
- CVS2: CV on drugs—random rows in Y (i.e., drugs) were blinded for testing.
- CVS3: CV on targets—random columns in Y (i.e., targets) were blinded for testing.

Under CVS1, we applied 5-repeats of stratified 10-fold cross-validation to evaluate the performance of MDADTI model. In each round, we used 90% of elements in Y as training data and the remaining 10% of elements as test data. Under CVS2, in each round, we used 90% of rows in Y as training data and the remaining 10% of rows as test data. Under CVS3, in each round, we used 90% of columns in Y as training data and the remaining 10% of columns as test data. These three settings CVS1, CVS2 and CVS3 refer to the DTIs prediction for 1) new (unknown) pairs, 2) new drugs, and 3) new targets, respectively.

For datasets GPCR, IC, E, and DrugBank_FDA, in order to determine the layer configurations of MDA (the number of layers and the number of neurons in each layer) in MDADTI model, we applied 5-repeats of stratified 10-fold cross-validation under CVS1 to evaluate the performance of MDADTI models with different layer configurations of MDA. Stratified 10-fold cross-validation can make the category ratio in each fold be consistent with that in the whole dataset.

For the small dataset NR, considering the overfitting problem on the small dataset of MDADTI model, for each CV setting, we applied transfer learning strategy (Pan and Yang, 2009) to predict DTIs. We first pretrained MDADTI model under CVS1 setting with the drug-target interactions in the E dataset. Then we froze all layers of the pretrained models except the output layer, i.e., only set weights of the output layer to be trainable. Finally, we finetuned the pretrained model with drug-target interactions data in NR dataset and predicted DTIs under CVS1. The transfer learning process under CVS2 and CVS3 settings are the same as that under CVS1 setting.

In order to focus on the differences between MDADTI and other methods on NR, GPCR, IC, E, and DrugBank_FDA datasets, we applied 5-repeats of 10-fold cross-validation under three different settings to compare the performance of MDADTI with DDR (Olayan et al., 2017), KronRLS-MKL(Nascimento et al., 2016), NRLMF(Liu et al., 2016), and BLM-NII (Mei et al., 2012).

### The Results of MDADTI With Different Layer Configurations of MDA

**Figure 3:**
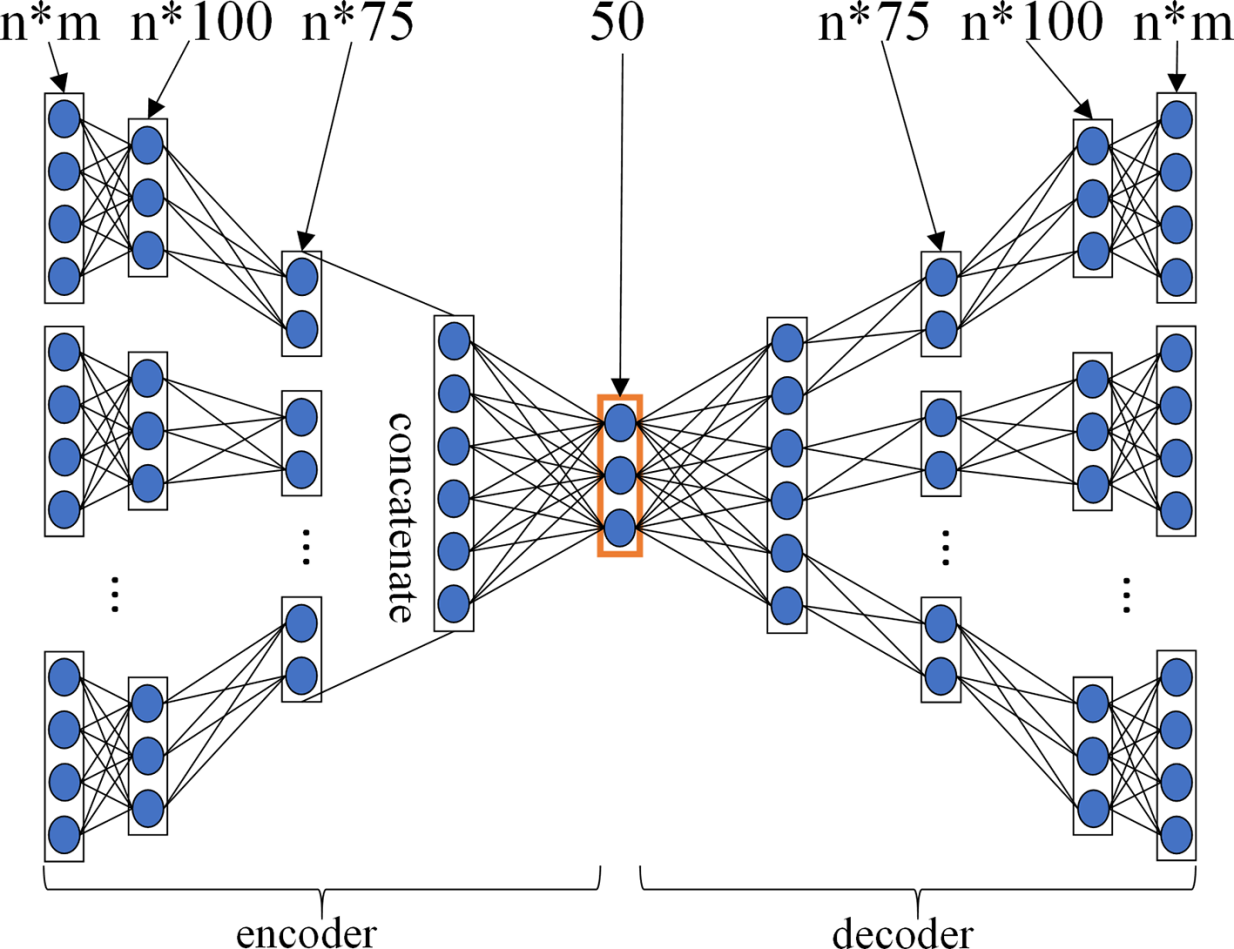
The layer configurations diagram of MDA. The layer configurations are [n*m, n*100, n*75, 50, n*75, n*100, n*m]. It consists of 7 layers of neurons, including 1 input layer n*m, where n is the number of input similarity measures, and m is the number of columns of each similarity matrix, i.e. the number of drugs (targets), 1 output layer n*m, 2 encoding layers n*100 and n*75, 2 decoding layers n*75 and n*100, 1 feature layer with 50 neurons.

For GPCR, IC, E, and DrugBank_FDA datasets, in order to determine the layer configurations of two MDAs for extracting drug and target features in the MDADTI model, we applied 5-repeats of 10-fold cross-validation under CVS1 to evaluate the performance of MDADTI models with different layer configurations of MDAs. **Figure 3** is a layer configurations diagram of MDA whose layer configurations is [n*m, n*100, n*75, 50, n*75, n*100, n*m]. It consists of 7 layers of neurons, including 1 input layer n*m, where n is the number of input similarity measures and m is the number of columns of each similarity matrix, i.e., the number of drugs (targets); 1 output layer n*m, 2 encoding layers n*100 and n*75, 2 decoding layers n*75 and n*100, and 1 feature layer with 50 neurons.

For each dataset, we input all similarity measures listed in **Table 2** into three different MDADTI models whose layer configurations of two MDAs are different, and then we trained them to predict DTIs. The performance of MDADTI models for different layer configurations of two MDAs in four datasets under 5-repeats of 10-fold cross-validation is provided in **Table 3**; *n*_d_ and *n*_t_ are the number of drugs and targets in each of the four datasets, respectively. The AUC and AUPR values in bold are highest among three sets of evaluation indicator values corresponding tree different layer configurations of MDAs.

**Table 3:** The comparison results of MDADTI models with different layer configurations of two MDAs under 5-repeats of 10-fold cross-validation on four datasets. The AUC and AUPR values in bold are highest among three sets of evaluation indicator values corresponding tree different layer configurations of MDAs in each dataset.

Datasets	Different layer configurations of MDAs		AUC	AUPR
GPCR	drug	[n*n_d_,50,n*n_d_]	**0.980**	**0.978**
	target	[n*n_t_,25,n*n_t_]		
	drug	[n*n_d_,n*75,50,n*75,n*n_d_]	0.965	0.963
	target	[n*n_t_,n*50,25,n*50,n*n_t_]		
	drug	[ n*n_d_,n*150,n*75,50,n*75,n*150,n*n_d_]	0.930	0.925
	target	[ n*n_t_,n*75,n*50,25,n*50,n*75,n*n_t_]		
IC	drug	[n*n_d_,50,n*n_d_]	**0.991**	**0.987**
	target	[ n*n_t_,50,n*n_t_]		
	drug	[n*n_d_,n*75,50,n*75,n*n_d_]	0.944	0.923
	target	[n*n_t_,n*75,50,n*75,n*n_t_]		
	drug	[ n*n_d_,n*150,n*75,50,n*75,n*150,n*n_d_]	0.914	0.906
	target	n*n_t_,n*150,n*75,50,n*75,n*150,n*n_t_]		
E	drug	[n*n_d_,100,n*n_d_]	0.956	0.947
	target	[n*n_t_,100,n*n_t_]		
	drug	[n*n_d_,n*200,100,n*200,n*n_d_]	**0.983**	**0.980**
	target	[n*n_t_,n*200,100,n*200,n*n_t_]		
	drug	[n*n_d_,n*300,n*200,100,n*200,n*300,n*n_d_]	0.893	0.886
	target	[n*n_t_,n*300,n*200,100,n*200,n*300,n*n_t_]		
DrugBank_FDA	drug	[n*n_d_,100,n*n_d_]	0.925	0.912
	target	[n*n_t_,100,n*n_t_]		
	drug	[n*n_d_,n*200,100,n*200,n*n_d_]	**0.963**	**0.959**
	target	[n*n_t_,n*200,100,n*200,n*n_t_]		
	drug	[n*n_d_,n*300,n*200,100,n*200,n*300,n*n_d_]	0.946	0.938
	target	[n*n_t_,n*300,n*200,100,n*200,n*300,n*n_t_]		

From **Table 3** we observed that for GPCR dataset, MDADTI achieved the highest AUC and AUPR when two MDAs have only one feature layer. Therefore, the MDA extracting the drug features is configured as [ n*n_*d*_,50,n*n_*d*_ ], and the MDA extracting target features is configured as [ n*n_*t*_,25,n*n_*t*_ ] when we applied MDADTI to predict DTIs in GPCR dataset. The AUC and AUPR of MDADTI are 0.980 and 0.978, respectively. For IC dataset, MDADTI achieved the highest AUC and AUPR when two MDAs have only one feature layer. Therefore, the MDA extracting drug features is configured as[ n*n_*d*_,50,n*n_*d*_ ], and the MDA extracting target features is configured as [ n*n_*t*_,50,n*n_*t*_ ] when we applied MDADTI to predict DTIs in IC dataset. The AUC and AUPR of MDADTI are 0.991 and 0.987, respectively. For E dataset, MDADTI achieved the highest AUC and AUPR when two MDAs have 1 encoding layer, 1 feature layer, and 1 decoding layer. Therefore, the MDA extracting drug features is configured as [ *n* * *n*_*d*_, *n* * 200, 100, *n* * 200, *n* * *n*_*d*_ ] and the MDA extracting target features is configured as [ *n* * *n*_*t*_, *n* * 200, 100, *n* * 200, *n* * *n*_*t*_ ] when we applied MDADTI to predict DTIs in E dataset. The AUC and AUPR of MDADTI are 0.983 and 0.980, respectively. For DrugBank_FDA dataset, MDADTI achieved the highest AUC and AUPR when two MDAs have 1 encoding layer, 1 feature layer, and 1 decoding layer. Therefore, the MDA extracting drug features are configured as [ n*n_*d*_,n*200,100,*n* * 200, *n* * *n*_*d*_ ]and the MDA extracting target features are configured as [ *n* * *n*_*t*_, *n* * 200 , 100, *n* * 200, *n* * *n*_*t*_ ] when we applied MDADTI to predict DTIs in DrugBank_FDA dataset. The AUC and AUPR of MDADTI are 0.963 and 0.959, respectively.

**Figure 4:**
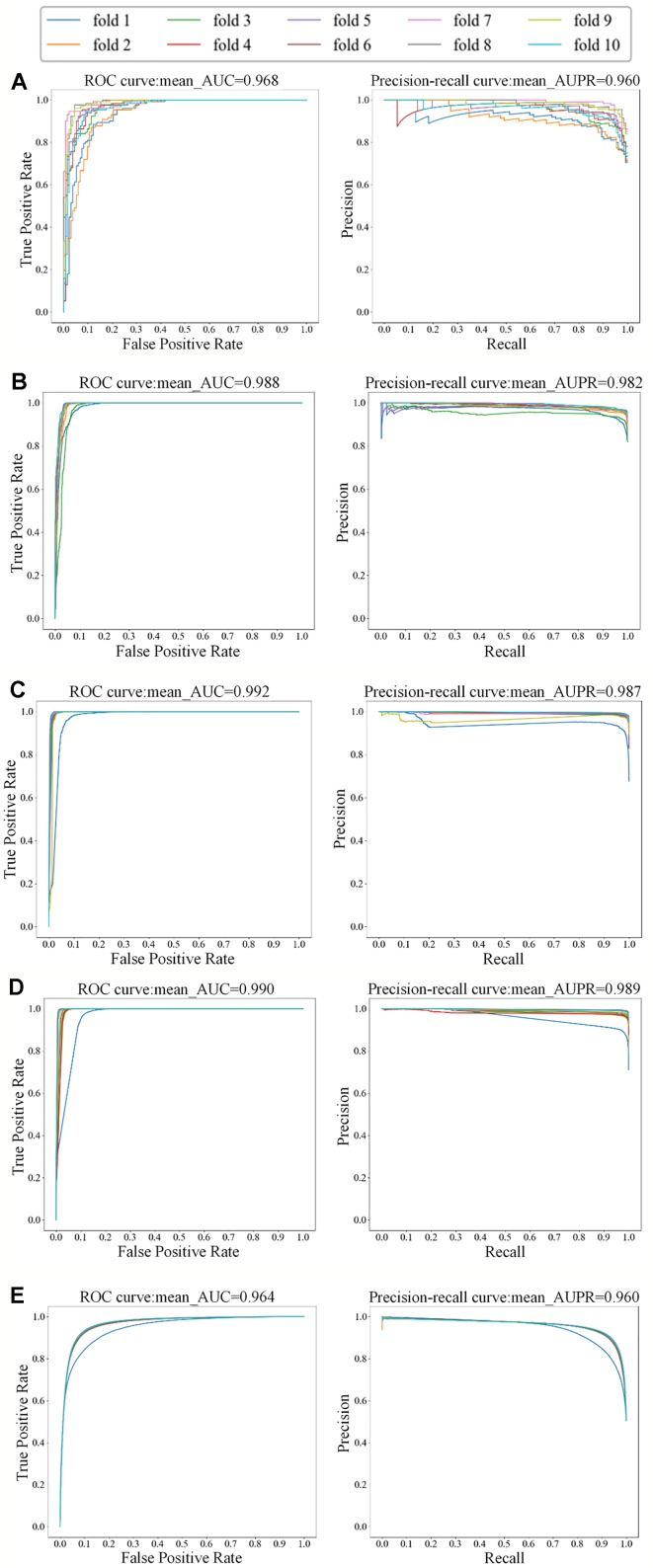
The ROC and precision-recall curves of the first repeat of 10-fold cross-validation for five datasets; the left is the ROC curve and the right is the precision-recall curve. **(A)** The ROC and precision-recall curves for NR dataset; **(B)** The ROC and precision-recall curves for GPCR dataset; **(C)** The ROC and precision-recall curves for IC dataset; **(D)** The ROC and precision-recall curves for E dataset; **(E)** The ROC and precision-recall curves for DrugBank_FDA dataset.

For the small dataset NR, we applied transfer learning strategy to predict DTIs, and also applied 5-repeats of 10-fold cross-validation to evaluate MDADTI and obtain AUC and AUPR. Finally, we obtained AUC and AUPR of MDADTI in NR, GPCR, IC, E, and DrugBank_FDA datasets. AUC are 0.966, 0.980, 0.991, 0.983, and 0.963, respectively; AUPR are 0.959, 0.978, 0.987, 0.980, and 0.959, respectively. **Figure 4** shows the ROC curve and precision-recall curve of the first repeat of 10-fold cross-validation in five datasets. The mean_AUC and mean_AUPR are the average AUC and average AUPR of MDADTI in the first repeat of 10-fold cross-validation. The train/valid accuracy-epoch and loss-epoch curves for each dataset are provided in **Figure S1** of **Supplementary Material** while selecting fold1 as the test set and the remaining as train set when we performed the first repeat of 10-fold cross-validation. From **Figure S1** we can observe that the change law of accuracy and loss of our model while validating is consistent with that while training, which demonstrates that overfitting has been effectively processed for each dataset. The hyperparameters of MDADTI model for each dataset under CVS1 setting are provided in **Table S1** of the **Supplementary Material**.

### Comparisons With Other Methods

In order to focus on the differences between MDADTI and other methods on NR, GPCR, IC, and E datasets under three different CV settings, we provided a detailed comparison with DDR, KronRLS-MKL, NRLMF, and BLM-NII methods. For DrugBank_FDA dataset, we only compared our method MDADTI with BLM-NII and NRLMF under three cross-validation settings because of the large amount of data in DrugBank_FDA dataset and the high-complexity of DDR and KronRLS-MKL methods, resulting in their longer runtime than our method.

DDR: First, it applied a similarity selection procedure to select a set of informative and less-redundant set of similarities for drugs and for target proteins. Then it manually extracted 12 different path-category-based feature matrices from the heterogeneous network, which consists of known drug-target interaction network and similarity networks for drugs and targets. Finally, it sent feature matrices to the Random Forest (RF) to predict DTIs.

KronRLS-MKL: First, it computed the weighted combination of multiple drug kernels and target kernels to get the final drug kernel and target kernel, then it computed the Kronecker product of final drug kernel and target kernel as the drug-target pairwise kernel. Finally, it applied Kronecker regularized least squares (KronRLS) to predict DTIs.

NRLMF: NRLMF represented the properties of a drug and a target as two latent vectors in the shared low dimensional latent space. For each drug-target pair, the interaction probability is modeled by a logistic function of the drug-specific and target-specific latent vectors. Moreover, the neighborhood regularization based on the drug similarities and target similarities is utilized to further improve the prediction ability of the model.

BLM-NII: BLM-NII integrated Bipartite Local Model (BLM) method with a neighbor-based interaction-profile inferring (NII) procedure to form a DTI prediction approach, where the RLS classifier with GIP kernel was used as the local model.

**Figure 5:**
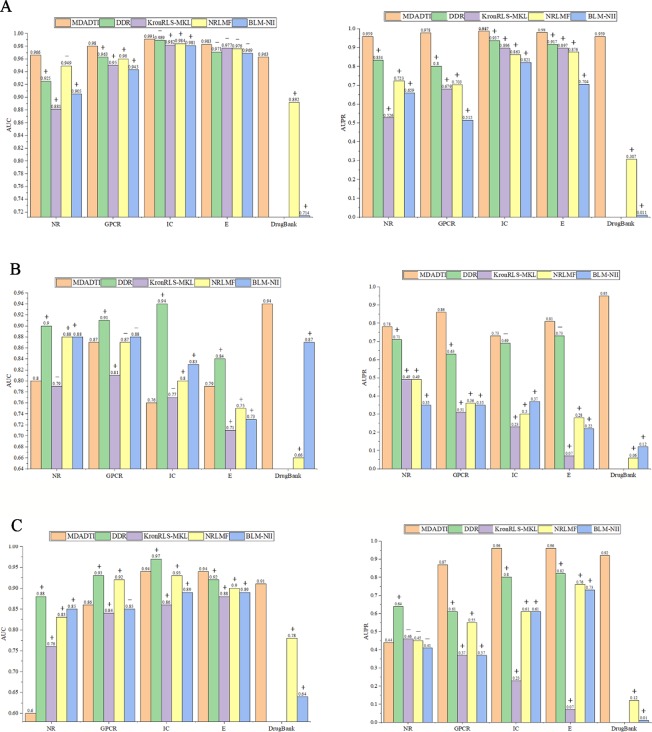
Comparison of AUC and AUPR among MDADTI, DDR, KronRLS-MKL, NRLMF, and BLM-NII methods on NR, GPCR, IC, E, and Drugbank_FDA datasets under CVS1, CVS2, and CVS3 setting. **(A)** Comparison of AUC and AUPR under CVS1 setting; **(B)** Comparison of AUC and AUPR under CVS2 setting; **(C)** Comparison of AUC and AUPR under CVS3 setting. The symbols +/- denote if the differences between our method MDADTI and other methods are statistically significant (+) or not (-) at the significance level of 0.05.

For comparison with these methods under CVS1 setting, we used 5-repeats of 10-fold cross-validation based on drug-target pairs to evaluate the predictive performance of DDR, KronRLS-MKL, NRLMF, and BLM-NII. **(Figure 5A)** shows the comparison of AUC and AUPR of MDADTI, DDR, KronRLS-MKL, NRLMF, and BLM-NII on five datasets under CVS1 setting. It can be seen from the figure that the performance of MDADTI has improved compared with the other methods. For NR, GPCR, IC, and E datasets, the growth rates of AUC of MDADTI compared to DDR, KronRLS-MKL, NRLMF, and BLM-NII are as follows: (NR: 4.43%, 9.65%, 1.79%, 6.74%), (GPCR: 1.77%, 3.16%, 2.08%, 3.81%), (IC: 0.20%, 0.92%, 0.71%, 1.02%), and (E: 1.03%, 0.61%, 0.72%, 1.34%). The growth rates of AUPR of MDADTI compared to DDR, KronRLS-MKL, NRLMF, and BLM-NII are as follows: (NR: 14.98%, 82.31%, 32.64%, 45.52%), (GPCR: 22.25%, 44.03%, 39.12%, 89.90%), (IC: 5.34%, 10.16%, 14.37%, 20.22%), and (E: 2.17%, 9.25%, 11.87%, 39.20%). For DrugBank_FDA dataset, we only compared our method with NRLMF and BLM-NII, and the growth rates of AUC of MDADTI compared to NRLMF and BLM-NII are 7.96% and 34.87%, respectively. In terms of AUPR, our method has improved by 213.40% than NRLMF that performs better between these two baselines methods.

The experimental results show that MDADTI is superior to DDR, KronRLS-MKL, NRLMF, and BLM-NII under CVS1 setting. The above comparison does not guarantee the efficacy and superiority of our proposed method. The possibility of getting good results by chance cannot be ignored. Thus, we performed paired t-test at significance level p = 0.05 to check if the differences between our method and the other methods are statistically significant or not under CVS1 setting. The specific details are as follows: we obtained 50 AUCs and 50 AUPRs for each method after performing five repeats of 10-fold cross-validation. In order to check if the differences between our method and each of baseline methods are statistically significant or not, i.e., check if mean AUCs (AUPRs) (mean AUC is the mean value of 50 AUCs) of them have significant differences, for each baseline method, we performed paired t-test based on 50 AUCs (AUPRs) of our method MDADTI and 50 AUCs (AUPRs) of the baseline method, respectively. We also combined bootstrap method to increase the sample size and used 2000 bootstrap samples for performing paired t-test.

The p-values of AUC and AUPR between our method and the other methods under CVS1 setting are reported in **Table S4(a)** of **Supplementary Material**, whereas p-values are less than 0.05 to demonstrate the statistical superiority of our method. For NR, GPCR, IC, and E dataset, in terms of AUC, from **Table S4(a)** we can observe that MDADTI outperforms the other baseline methods, being statistically significant in most cases at the significance level of 0.05, except one comparison case with DDR in IC dataset, one comparison case with NRLMF in NR dataset, and four comparison cases with the other four competing methods in E dataset. In terms of AUPR, we can observe that MDADTI outperforms other baseline methods, being statistically significant in all cases at the significance level of 0.05. For DrugBank_FDA dataset, in terms of AUC, we can see that our method performs best, and it outperforms NRLMF and BLM-NII methods, being statistically significant at the significance level of 0.05. In terms of AUPR, we can see that our method also performs best, and it outperforms NRLMF and BLM-NII methods, being statistically significant.

For comparison with these methods under CVS2 and CVS3 setting, we used 5-repeats of 10-fold cross-validation based on drugs and targets to evaluate the predictive performance of MDADTI, DDR, KronRLS-MKL, NRLMF and BLM-NII. The hyperparameters of MDADTI model for each dataset under CVS2 and CVS2 settings are provided in **Table S2** and **Table S3** of **Supplementary Material**, respectively. The comparison of AUC and AUPR among MDADTI, DDR, KronRLS-MKL, NRLMF, and BLM-NII methods on NR, GPCR, IC, E, and DrugBank_FDA datasets under CVS2 and CVS3 settings are provided in **Figure 5B** and **Figure 5C**. The screenshot of MDADTI when it predicts DTIs in GPCR dataset under CVS2 setting is provided in **Figure S2** of **Supplementary Material**. The program flow chart of the code of MDADTI under CVS1,CVS2,and CVS3 settings is provided in **Figure S3** of **Supplementary Material**.

From **Figure 5B** we can see that under CVS2 setting in NR, GPCR, IC, and E datasets, for AUPR, the performance of MDADTI is improved on these four datasets, and the growth rates are 9.86%, 36.51%, 5.80%, 10.96%, respectively, compared with the best method DDR among the baseline methods. For AUC, compared with these four methods, MDADTI performed better on E dataset, which was 11.26%, 5.33%, and 8.22% higher than KronRLS-MKL, NRLMF, and BLM-NII, respectively; MDADTI performed moderately on the other three datasets. For DrugBank_FDA dataset, our method performed better than NRLMF and BLM-NII methods in both AUC and AUPR.

Under CVS3 setting, from **Figure 5C** we can see that for NR, GPCR, IC, and E datasets, the AUPR of MDADTI has a certain improvement in GPCR, IC, and E datasets. The AUPR has increased by 42.62%, 20%, and 17.07% in GPCR, IC, and E datasets, respectively, compared with the best performing method DDR; for AUC, the AUC of MDADTI is the highest in E dataset compared with the other methods, which was increased by 2.17% than the AUC of DDR. For IC dataset, the AUC of MDADTI was increased by 9.30%, 1.08%, and 5.62% compared with KronRLS-MKL, NRLMF, and BLM-NII, respectively. Our method performed moderately in the GPCR dataset, but our method performed poorly on NR dataset. After analysis, it is found that the data volume of the E dataset is 295480 and the large amount of samples make deep learning model perform better; however, the data volume of the NR dataset is only 1404, which is relatively small and does not meet the requirements for data volume of deep learning. Although we applied transfer learning method to predict DTIs of NR datasets under CVS3 setting, the train set of CVS3 setting contains relatively little information, which affects the effect of transfer learning and leads to poor prediction results. For DrugBank_FDA dataset, our method performed better than NRLMF and BLM-NII methods in both AUC and AUPR.

As a kind of data-driven method, deep learning methods are superior to traditional machine learning methods when the amount of data is quite large. By comparing the performance of our method on the five datasets, our method performed best on E and DrugBank_FDA datasets and performed worst on NR dataset, which is consistent with the theory of deep learning.

Similar to CVS1 setting, we performed paired t-test at significance level p = 0.05 to check if the differences between our method and the other methods are statistically significant or not under CVS2 and CVS3 settings. The p-values of AUC and AUPR between our method and the other methods under CVS2 and CVS3 settings are tabulated in **Table S4(b)** and **Table S4(c)** of **Supplementary Material**, respectively.

For CVS2 setting, in terms of AUC, from **Table S4(b)** we can see that our method outperforms KronRLS-MKL in GPCR dataset, being statistically significant at the significance level of 0.05, and it also outperforms KronRLS-MKL, NRLMF, and BLM-NII in E dataset. For DrugBank_FDA dataset, our method MDADTI performs best compared with NRLMF and BLM-NII methods, and it outperforms them, being statistically significant. In terms of AUPR, from **Table S4(b)** we can see that our method performs best in five datasets and it outperforms other baseline methods, being statistically significant in most cases at the significance level of 0.05, except two comparison cases with DDR in IC and E datasets.

For CVS3 setting, in terms of AUC, from **Table S4(c)** we can see that our method outperforms KronRLS-MKL method in GPCR dataset, being statistically significant at the significance level of 0.05. Our method also outperforms KronRLS-MKL, NRLMF, and BLM-NII methods in IC dataset, being statistically significant. For E and DrugBank_FDA datasets, our method outperforms all baseline methods, being statistically significant at the significance level of 0.05. In terms of AUPR, from **Table S4(c)** we can see that our method MDADTI performs best in GPCR, IC, E, and DrugBank_FDA datasets, and it outperforms all baseline methods, being statistically significant in all cases. The comparison of AUC and AUPR between MDADTI with transfer learning and MDADTI without transfer learning on NR dataset is reported in **Table S6** of **Supplementary Material**. The performance of MDADTI with SMOTE method and MDADTI without SMOTE method is reported in **Table S7** of **Supplementary Material**. 

All above analyses demonstrate that MDADTI is superior to DDR, KronRLS-MKL, NRLMF, and BLM-NII. The main reason is that different from DDR, KronRLS-MKL, NRLMF, and BLM-NII, which directly took the original multiple similarity measures as input and manually extracted the features of the drug-target pairs, MDADTI applied RWR method and PPMI to capture the global structure information of the similarity measures, and applied the multi-layer nonlinear functions of MDA to capture the complex non-linear relationship among features, and automatically learned the deep feature representation of drugs and targets, which are helpful to improve the predictive performance.

For large datasets GPCR, IC, and E, MDA reduced the dimension of drug feature and target feature while automatically learning them. The dimension of drug feature in GPCR dataset is reduced from 223 to 50, and the dimension of target feature is reduced from 95 to 25. The dimension of drug feature and target feature in IC dataset are reduced from 210 and 204 to 50, respectively. The dimension of drug feature and target feature in E dataset are reduced from 1482 and 1408 to 100, respectively. Dimensionality reduction accelerates the training speed and saves the time costs running on large datasets of MDADTI model.

We observed that the predictive performance of MDADTI is greatly improved in NR dataset under CVS1 setting, which indicates that our transfer learning strategy helps MDADTI achieve superior performance with a small amount of labeled data. This is because we used DTIs in E datasets to pretrain MDADTI model, and froze all layers except the output layer of the pretrained model, that is, set the parameters of these frozen layers to be untrainable. These parameters contain the knowledge learned from the E dataset, which are also applicable to the NR dataset. Therefore, our transfer learning strategy can make MDADTI predict DTIs more accurately on small datasets.

According to statistics, about one-third of the small molecule drugs in the world drug market are activators or antagonists of GPCRs, which are related to many diseases, and GPCR is the target of about 40% of modern drugs (Marinissen and Gutkind, 2001). MDADTI has a significant improvement in predictive performance on GPCR datasets under CVS1 setting. Therefore, MDADTI can be used as an effective tool to predict GPCR target and has great significance for drug development and disease treatment.

### Effectiveness of Feature Learning for Drugs and Targets With MDA

In order to evaluate the effectiveness of feature learning for drugs and targets with MDA for improving the predictive performance of MDADTI, we designed another RWR_DNN method to be compared with MDADTI. Firstly, RWR_DNN takes multiple

similarity measures for drugs (targets) as original inputs. Then, it uses RWR and PPMI method to calculate multiple topological similarity matrices for drugs (targets). Next, it averages multiple topological similarity matrices of drugs (targets) to form the feature of drugs (targets). Finally, the features of drugs and targets are concatenated together and sent into DNN for predicting DTIs. Similarly, we applied 5-repeats of 10-fold cross-validation under CVS1, CVS2, and CVS3 setting to evaluate the performance of RWR_DNN. The hyperparameters of RWR_DNN method on NR, GPCR, IC, and E dataset under three settings are the same with that of MDADTI, which are reported in **Table S1**–**S3** of the **Supplementary Material**.

**Figure 6:**
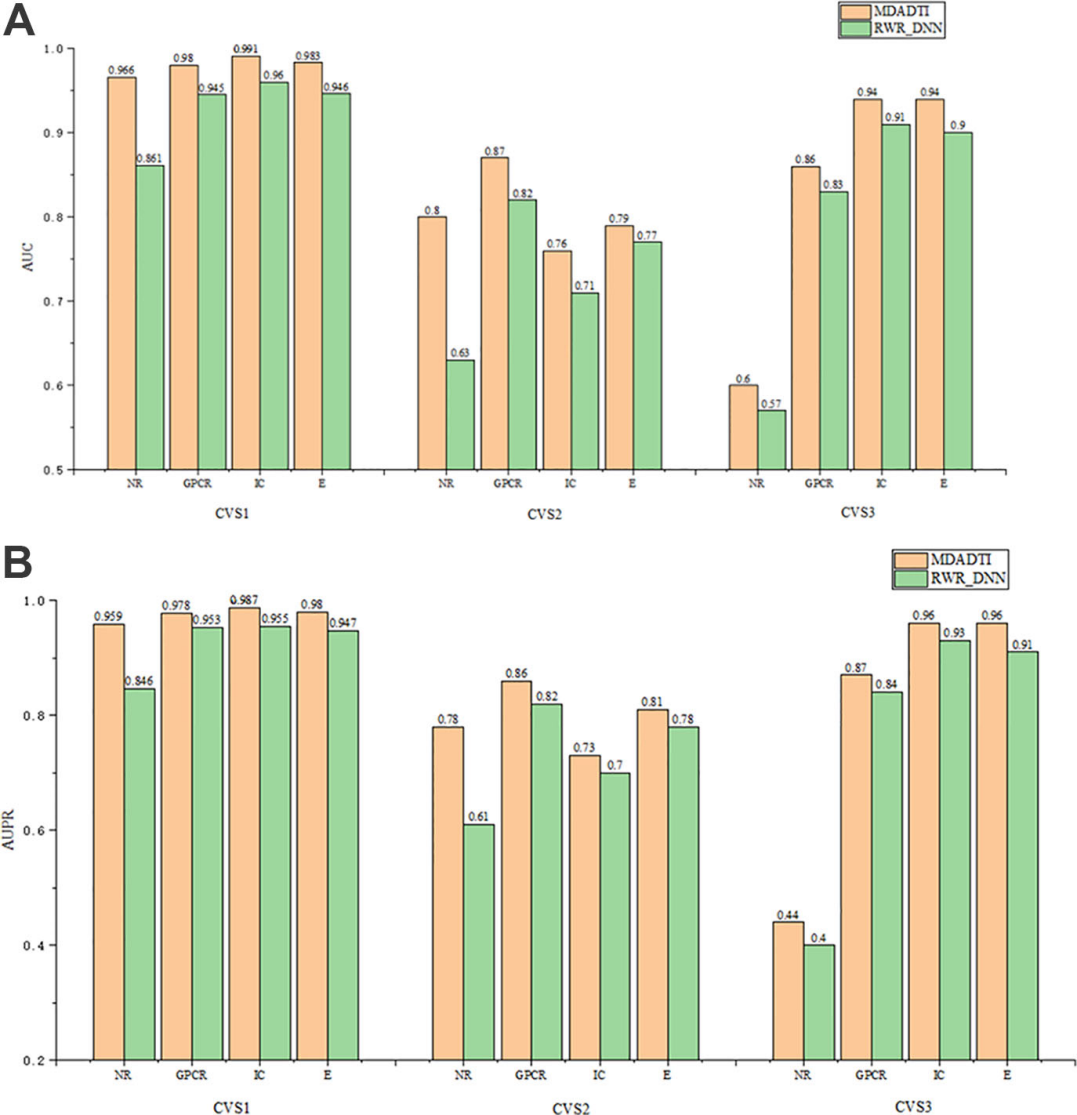
The comparison of AUC and AUPR between MDADTI and RWR_DNN method on NR, GPCR, IC and E dataset under CVS1, CVS2 and CVS3 setting. **(A)** Comparison of AUC **(B)** Comparison of AUPR.

The comparison results of RWR_DNN and MDADTI on NR, GPCR, IC, and E datasets in 5-repeats of 10-fold cross-validation are shown in **Figure 6**, where **Figure 6 (A)** is the comparison of AUC and **Figure 6 (B)** is the comparison of AUPR. We can see the AUC and AUPR values of MDADTI are higher than that of RWR_DNN in all cases. The results demonstrate that MDA can automatically learn deep feature representations of drugs and targets from multiple topological similarity matrices and effectively improve the predictive performance of MDADTI method.

### Prediction and Validation of Unknown DTIs

In this paper, we used NR, GPCR, IC, E, and DrugBank_FDA datasets to evaluate the performance of our proposed method MDADTI, and for each dataset, we used 5-repeats of 10-fold cross-validation to evaluate the performance of MDADTI method. Since the negative samples in the NR, GPCR, IC, E and DrugBank_FDA datasets are unknown DTIs, we evaluated the practical ability of MDADTI model in predicting new (unknown) interactions. New interactions are predicted high-probability drug-target pairs, but they are unknown DTIs in NR, GPCR, IC, E, and DrugBank_FDA datasets.

In order to implement this, we used the trained model to predict unknown DTIs in each dataset and output the interaction probability of a drug-target pair. Then we ranked them in descending order according to the predicted probability. Finally, we selected the top 100 predicted unknown DTIs and validated them in six reference databases, i.e., to check if they are included in any of six reference databases: ChEMBL (Gaulton et al., 2011), DrugBank (Knox et al., 2010), KEGG (Kanehisa et al., 2011), Matador (Günther et al., 2007), CTD (Davis et al., 2016), and STITCH (Kuhn et al., 2007). These six reference databases are online databases that include a large number of proved known DTIs and they are used by related literature to evaluate the actual ability of their methods in predicting unknown DTIs (Liu et al., 2016; Nascimento et al., 2016; Olayan et al., 2017).

**Table 4** shows the top 30 unknown interactions predicted by the MDADTI model on E dataset. In this table, DTIs in bold indicate that they exist in one or more reference databases, and the third column shows their predicted probability. For each drug-target pair, the reference databases containing it are displayed in the last column of the table, where C is the abbreviation of ChEMBL, D is the abbreviation of DrugBank, M is the abbreviation of Matador, K is the abbreviation of KEGG, T is the abbreviation of CTD, and S is the abbreviation of STITCH. For example, the DTI ranking No. 1 is D00528, hsa1549 and its predicted interaction probability is 1.0, which is validated in the Matador database. It can be seen from the table that 21 out of 30 unknown interactions are validated in at least one of the six reference databases.

**Table 4:** Top 30 unknown DTIs predicted by MDADTI model on E dataset. DTIs in bold indicate that they are validated in one or more reference databases.

Rank	Drug	Target	Probability	Databases					
**1**	**D00528**	**hsa1549**	**1.0**				**M**		
**2**	**D00542**	**hsa1571**	**0.9997**				**M**		
**3**	**D00501**	**hsa5150**	**0.9997**	**C**					
**4**	**D00437**	**hsa1559**	**0.9997**				**M**		
**5**	**D00043**	**hsa11330**	**0.9995**				**M**		
**6**	**D00528**	**hsa5150**	**0.9992**		**D**	**K**			
**7**	**D00410**	**hsa1543**	**0.9988**				**M**		
**8**	**D00691**	**hsa8564**	**0.9985**						**S**
**9**	**D00437**	**hsa1585**	**0.9981**				**M**		
**10**	**D00410**	**hsa1585**	**0.9981**				**M**		
**11**	**D00139**	**hsa1543**	**0.9972**				**M**		
**12**	**D00043**	**hsa2147**	**0.9965**				**M**		
**13**	**D01441**	**hsa5594**	**0.9884**					**T**	
**14**	**D00126**	**hsa246**	**0.9869**				**M**		
15	D00043	hsa1504	0.977						
**16**	**D00217**	**hsa1559**	**0.9683**					**T**	
**17**	**D01223**	**hsa3988**	**0.9644**				**M**		
**18**	**D00038**	**hsa5742**	**0.9640**					**T**	
19	D01223	hsa5538	0.9616						
20	D00002	hsa31	0.9553						
**21**	**D01441**	**hsa1021**	**0.9546**					**T**	
**22**	**D00528**	**hsa5743**	**0.9467**					**T**	
23	D00139	hsa5742	0.9344						
**24**	**D00217**	**hsa1558**	**0.9338**					**T**	
**25**	**D00043**	**hsa1636**	**0.9326**				**M**		
26	D00002	hsa7298	0.9207						
27	D03670	hsa1579	0.8932						
28	D01441	hsa3551	0.8806						
**29**	**D00097**	**hsa5743**	**0.8787**		**D**		**M**		
30	D00043	hsa686	0.8688						

**Figure 7:**
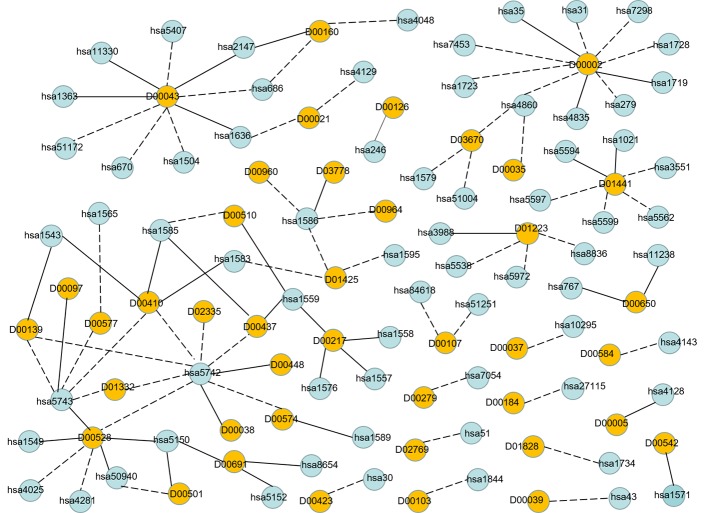
Network visualization of the top 100 unknown DTIs in E dataset. Yellow and blue nodes represent drugs and targets, respectively. Solid lines represent verified interaction and dashed lines represent unverified interactions. There are 40 unknown DTIs that were verified.

In order to visualize the validation of unknown DTIs more intuitively, we visualized 100 high-probability unknown DTIs in E dataset. **Figure 7** is the network visualization of the top 100 unknown DTIs in E dataset predicted by MDADTI model. Yellow and blue nodes represent drugs and targets, respectively. Solid lines represent verified interactions while dashed lines represent unverified interactions. It can be seen from the figure that there are potential interactions between a drug and multiple targets, and some of them have been verified in six reference databases. For example, 33.33% (3/10) of the potential targets of drug D00002 have been verified in reference databases; D00002 represents nicotinamide adenine dinucleotide (NADH), which is widely used in many diseases like tuberculosis, Alzheimer's, and Parkinson disease. 44.44% (4/9) of the potential targets of D00043 are validated in the reference databases, and D00043 represents isofluorphate, a powerful miotic used mainly in the treatment of glaucoma. 60% (3/5) of the potential targets of drug D00410 are validated in reference databases, and D00410 represents metyrapone, an inhibitor of the enzyme steroid 11-beta-monooxygenase, which is used as a test of the feedback hypothalamic-pituitary mechanism in the diagnosis of Cushing syndrome. 57.14% (4/7) of the potential targets of drug D00528 are verified in the reference database. We also observed that a target may interact with multiple drugs, and some of them are verified in six reference databases. For example, hsa1559 interacts with D00510, D00217, and D00437 at the same time, and all of them are verified in six reference databases. hsa5150 interacts with D00528, D00501, and D00691, and all of them are verified in six reference databases.

Finally, **Table 5** summarizes the validated proportion of top N unknown DTIs (N = 10, 30, 50, 100) on five datasets. The validated proportion of top 10 unknown DTIs are 50%, 80%, 80%, 100%, 80%, respectively. The fractions of validated DTIs of MDADTI, NRLMF, BLM-NII among the predicted Top N(N = 10, 30, 50) DTIs in NR,GPCR,IC, and E datasets are provided in **Table S5**. The fractions of validated DTIs of NRLMF, BLM-NII are provided by (Liu et al., 2016). Since these databases are still being updated, the proportion of new DTIs predicted by MDADTI model will increase in the future. All the above analyses proved that MDADTI can effectively predict unknown DTIs because MDADTI model integrated multiple similarity measures of drugs and targets, which provides abundant information for predicting DTIs. Moreover, MDADTI not only considered the original information of similarity measure but also captured the global structure information of similarity measures, which improved the prediction accuracy of DTIs. The most important reason is that MDADTI applied MDA to automatically learn the deep representation of drug feature and target feature from multiple topological similarity matrices of drugs and targets, which contributes to the effective prediction of unknown DTIs.

**Table 5:** The fractions of true DTIs among the predicted Top N (N = 10, 30, 50,100) unknown DTIs in five datasets.

Dataset	Top N	Fraction
NR	Top10	50.00%
	Top30	43.33%
	Top50	28.00%
	Top100	20.00%
GPCR	Top10	80.00%
	Top30	66.67%
	Top50	60.00%
	Top100	40.00%
IC	Top10	80.00%
	Top30	50.00%
	Top50	40.00%
	Top100	32.00%
E	Top10	100.00%
	Top30	73.33%
	Top50	52.00%
	Top100	40.00%
DrugBank_FDA	Top10	80.00%
	Top30	66.67%
	Top50	68.00%
	Top100	46.00%

## Discussion

We proposed a novel method MDADTI to predict DTIs based on MDA. Compared with existing methods, MDADTI applied RWR and PPMI to calculate the topological similarity matrices of drugs and targets, capturing the global structure information of the similarity measures. Then MDA was applied to fuse multiple topological similarity matrices and learn the feature of drugs and targets while capturing the non-linear relationship among features. In addition, MDA also reduced the dimension of the feature of drugs and targets, which speeded up the training of MDADTI. To evaluate the performance of MDADTI, we compared MDADTI with DDR, KronRLS-MKL, NRLMF, and BLM-NII under three different cross-validation settings. The results showed that MDADTI achieved higher AUC and AUPR in five datasets than the other four methods under CVS1 setting. The predictive performance of MDADTI was greatly improved especially in GPCR and NR datasets. For CVS2 and CVS3 settings, our method has a great improvement in AUPR in five datasets, and it performed better in large datasets, like E and DrugBank_FDA datasets. These results proved that MDADTI is better than the other four baseline methods in predicting DTIs.

In addition, we evaluated the actual ability of MDADTI method to predict new interactions. For each dataset, we applied the trained MDADTI model to predict unknown interactions and selected the top 100 predictions to validate them in the six reference databases: ChEMBL, DrugBank, KEGG, Matador, STITCH, and CTD. The results showed that MDADTI method can effectively identify unknown DTIs.

Since our method currently only predicts whether there is an interaction between a drug and a target, we plan to predict the binding affinity scores for drug-target pairs in the next step.

## Data Availability Statement

All datasets generated for this study are included in the article/**Supplementary Material**.

## Author Contributions

HW and JW performed the majority of the analysis and primarily wrote the manuscript. CD performed some analysis and provided biological expertise. ZY performed some analysis of data and helped conceive the project. YL and DL completed the drawing of the charts in the results analysis and the layout of the manuscripts. All authors edited and approved the manuscript.

## Funding

This study was supported by research grants from the National Key Research and Development Plan of China (2018YFD0100204), the National Natural Science Foundation of China (61672374), National Natural Science Foundation of China (61976150), Key Research and Development Plan of Shanxi Province (201903D121151) and the Scientific and Technological Project of Shanxi Province (No. 201603D22103-2).

## Conflict of Interest

The authors declare that the research was conducted in the absence of any commercial or financial relationships that could be construed as a potential conflict of interest.

## References

[B1] Alanis-Lobato G. Andrade-Navarro M. A. Schaefer M. H. (2016). HIPPIE v2. 0: enhancing meaningfulness and reliability of protein–protein interaction networks. Nucleic Acids Res. 45, D408–D414. 10.1093/nar/gkw985 27794551 PMC5210659

[B2] Ba-Alawi W. Soufan O. Essack M. Kalnis P. Bajic V. B. (2016). DASPfind: new efficient method to predict drug–target interactions. J. Cheminformatics 8 (1), 15. 10.1186/s13321-016-0128-4 PMC4793623 26985240

[B3] Bleakley K. Yamanishi Y. (2009). Supervised prediction of drug–target interactions using bipartite local models. Bioinformatics 25 (18), 2397–2403. 10.1093/bioinformatics/btp433 19605421 PMC2735674

[B4] Boutet E. Lieberherr D. Tognolli M. Schneider M. Bansal P. Bridge A. J. (2016). “UniProtKB/Swiss-Prot, the manually annotated section of the UniProt KnowledgeBase: how to use the entry view,” in Plant Bioinformatics (Springer), 23–54. 10.1007/978-1-4939-3167-5_2 26519399

[B5] Campillos M. Kuhn M. Gavin A.-C. Jensen L. J. Bork P. (2008). Drug target identification using side-effect similarity. Science 321 (5886), 263–266. 10.1126/science.1158140 18621671

[B6] Cao S. Lu W. Xu Q. (2016). Deep neural networks for learning graph representations. In *Thirtieth AAAI Conference on Artificial Intelligence* p. 1145–1152.

[B7] Chawla N. V. Bowyer K. W. Hall L. O. Kegelmeyer W. P. (2002). SMOTE: synthetic minority over-sampling technique. J. Artif. Intell. Res. 16, 321–357. 10.1613/jair.953

[B8] Chen Y. Li Y. Narayan R. Subramanian A. Xie X. (2016). Gene expression inference with deep learning. Bioinformatics 32 (12), 1832–1839. 10.1093/bioinformatics/btw074 26873929 PMC4908320

[B9] Davis A. P. Grondin C. J. Johnson R. J. Sciaky D. King B. L. McMorran R. (2016). The comparative toxicogenomics database: update 2017. Nucleic Acids Res. 45 (D1), D972–D978. 10.1093/nar/gkw838 27651457 PMC5210612

[B10] Deng L. Fan C. Zeng Z. (2017). A sparse autoencoder-based deep neural network for protein solvent accessibility and contact number prediction. BMC Bioinf. 18 (16), 569. 10.1186/s12859-017-1971-7 PMC5751690 29297299

[B11] Fan X.-N. Zhang S.-W. Zhang S.-Y. Zhu K. Lu S. (2019). Prediction of lncRNA-disease associations by integrating diverse heterogeneous information sources with RWR algorithm and positive pointwise mutual information. BMC Bioinf. 20 (1), 87. 10.1186/s12859-019-2675-y PMC6381749 30782113

[B12] Fang H. Gough J. (2013). A disease-drug-phenotype matrix inferred by walking on a functional domain network. Mol. Biosyst. 9 (7), 1686–1696. 10.1039/c3mb25495j 23462907

[B13] Fu L. Peng Q. (2017). A deep ensemble model to predict miRNA-disease association. Sci. Rep. 7 (1), 14482. 10.1038/s41598-017-15235-6 29101378 PMC5670180

[B16] Gönen M. (2012). Predicting drug–target interactions from chemical and genomic kernels using Bayesian matrix factorization. Bioinformatics 28 (18), 2304–2310. 10.1093/bioinformatics/bts360 22730431

[B17] Günther S. Kuhn M. Dunkel M. Campillos M. Senger C. Petsalaki E. (2007). SuperTarget and Matador: resources for exploring drug-target relationships. Nucleic Acids Res. 36 (suppl_1), D919–D922. 10.1093/nar/gkm862 17942422 PMC2238858

[B14] Gaulton A. Bellis L. J. Bento A. P. Chambers J. Davies M. Hersey A. (2011). ChEMBL: a large-scale bioactivity database for drug discovery. Nucleic Acids Res. 40 (D1), D1100–D1107. 10.1093/nar/gkr777 21948594 PMC3245175

[B15] Gligorijević V. Barot M. Bonneau R. (2018). deepNF: deep network fusion for protein function prediction. Bioinformatics 34 (22), 3873–3881. 10.1093/bioinformatics/bty440 29868758 PMC6223364

[B18] Hao M. Bryant S. H. Wang Y. (2017). Predicting drug-target interactions by dual-network integrated logistic matrix factorization. Sci. Rep. 7, 40376. 10.1038/srep40376 28079135 PMC5227688

[B19] Hizukuri Y. Sawada R. Yamanishi Y. (2015). Predicting target proteins for drug candidate compounds based on drug-induced gene expression data in a chemical structure-independent manner. BMC Med. Genomics 8 (1), 82. 10.1186/s12920-015-0158-1 26684652 PMC4683716

[B20] Jacob L. Vert J.-P. (2008). Protein-ligand interaction prediction: an improved chemogenomics approach. Bioinformatics 24 (19), 2149–2156. 10.1093/bioinformatics/btn409 18676415 PMC2553441

[B27] Köhler S. Bauer S. Horn D. Robinson P. N. (2008). Walking the interactome for prioritization of candidate disease genes. Am. J. Hum. Genet. 82 (4), 949–958. 10.1016/j.ajhg.2008.02.013 18371930 PMC2427257

[B22] Kanehisa M. Goto S. Sato Y. Furumichi M. Tanabe M. (2011). KEGG for integration and interpretation of large-scale molecular data sets. Nucleic Acids Res. 40 (D1), D109–D114. 10.1093/nar/gkr988 22080510 PMC3245020

[B21] Kanehisa M. Furumichi M. Tanabe M. Sato Y. Morishima K. (2016). KEGG: new perspectives on genomes, pathways, diseases and drugs. Nucleic Acids Res. 45 (D1), D353–D361. 10.1093/nar/gkw1092 27899662 PMC5210567

[B23] Keiser M. J. Setola V. Irwin J. J. Laggner C. Abbas A. I. Hufeisen S. J. (2009). Predicting new molecular targets for known drugs. Nature 462 (7270), 175. 10.1038/nature08506 19881490 PMC2784146

[B24] Khan M. Hayat M. Khan S. A. Iqbal N. (2017). Unb-DPC: Identify mycobacterial membrane protein types by incorporating un-biased dipeptide composition into Chou's general PseAAC. J. Theor. Biol. 415, 13–19. 10.1016/j.jtbi.2016.12.004 27939596

[B25] Klabunde T. (2007). Chemogenomic approaches to drug discovery: similar receptors bind similar ligands. Br. J. Pharmacol. 152 (1), 5–7. 10.1038/sj.bjp.0707308 17533415 PMC1978276

[B26] Knox C. Law V. Jewison T. Liu P. Ly S. Frolkis A. (2010). DrugBank 3.0: a comprehensive resource for ‘omics' research on drugs. Nucleic Acids Res. 39 (suppl_1), D1035–D1041. 10.1093/nar/gkq1126 21059682 PMC3013709

[B29] Kuhn M. von Mering C. Campillos M. Jensen L. J. Bork P. (2007). STITCH: interaction networks of chemicals and proteins. Nucleic Acids Res. 36 (suppl_1), D684–D688. 10.1093/nar/gkm795 18084021 PMC2238848

[B28] Kuhn M. Letunic I. Jensen L. J. Bork P. (2015). The SIDER database of drugs and side effects. Nucleic Acids Res. 44 (D1), D1075–D1079. 10.1093/nar/gkv1075 26481350 PMC4702794

[B30] Lim H. Gray P. Xie L. Poleksic A. (2016). Improved genome-scale multi-target virtual screening *via a* novel collaborative filtering approach to cold-start problem. Sci. Rep. 6, 38860. 10.1038/srep38860 27958331 PMC5153628

[B31] Liu Y. Wu M. Miao C. Zhao P. Li X.-L. (2016). Neighborhood regularized logistic matrix factorization for drug-target interaction prediction. PloS Comput. Biol. 12 (2), e1004760. 10.1371/journal.pcbi.1004760 26872142 PMC4752318

[B32] Lounkine E. Keiser M. J. Whitebread S. Mikhailov D. Hamon J. Jenkins J. L. (2012). Large-scale prediction and testing of drug activity on side-effect targets. Nature 486 (7403), 361. 10.1038/nature11159 22722194 PMC3383642

[B33] Lu Y. Guo Y. Korhonen A. (2017). Link prediction in drug-target interactions network using similarity indices. BMC Bioinf. 18 (1), 39. 10.1186/s12859-017-1460-z PMC5240398 28095781

[B34] Marinissen M. J. Gutkind J. S. (2001). G-protein-coupled receptors and signaling networks: emerging paradigms. Trends In Pharmacol. Sci. 22 (7), 368–376. 10.1016/S0165-6147(00)01678-3 11431032

[B35] Mei J.-P. Kwoh C.-K. Yang P. Li X.-L. Zheng J. (2012). Drug–target interaction prediction by learning from local information and neighbors. Bioinformatics 29 (2), 238–245. 10.1093/bioinformatics/bts670 23162055

[B37] Núñez S. Venhorst J. Kruse C. G. (2012). Target–drug interactions: first principles and their application to drug discovery. Drug Discovery Today 17 (1-2), 10–22. 10.1016/j.drudis.2011.06.013 21777691

[B36] Nascimento A. C. Prudêncio R. B. Costa I. G. (2016). A multiple kernel learning algorithm for drug-target interaction prediction. BMC Bioinf. 17 (1), 46. 10.1186/s12859-016-0890-3 PMC4722636 26801218

[B38] Olayan R. S. Ashoor H. Bajic V. B. (2017). DDR: efficient computational method to predict drug–target interactions using graph mining and machine learning approaches. Bioinformatics 34 (7), 1164–1173. 10.1093/bioinformatics/btx731 PMC5998943 29186331

[B39] Pan S. J. Yang Q. (2009). A survey on transfer learning. IEEE Trans. Knowl. Data Eng. 22 (10), 1345–1359. 10.1109/TKDE.2009.191

[B40] Pan X. Fan Y.-X. Yan J. Shen H.-B. (2016). IPMiner: hidden ncRNA-protein interaction sequential pattern mining with stacked autoencoder for accurate computational prediction. BMC Genomics 17 (1), 582. 10.1186/s12864-016-2931-8 27506469 PMC4979166

[B41] Peng J. Zhang X. Hui W. Lu J. Li Q. Liu S. (2018). Improving the measurement of semantic similarity by combining gene ontology and co-functional network: a random walk based approach. BMC Syst. Biol. 12 (2), 18. 10.1186/s12918-018-0539-0 29560823 PMC5861498

[B42] Perlman L. Gottlieb A. Atias N. Ruppin E. Sharan R. (2011). Combining drug and gene similarity measures for drug-target elucidation. J. Comput. Biol. 18 (2), 133–145. 10.1089/cmb.2010.0213 21314453

[B43] Srivastava N. Hinton G. Krizhevsky A. Sutskever I. Salakhutdinov R. (2014). Dropout: a simple way to prevent neural networks from overfitting. J. Mach. Learn. Res. 15 (1), 1929–1958. 10.5555/2627435.2670313

[B44] Sutskever I. Martens J. Dahl G. Hinton G. (2013). On the importance of initialization and momentum in deep learning In *International conference on machine learning* p. 1139–1147.

[B45] van Laarhoven T. Nabuurs S. B. Marchiori E. (2011). Gaussian interaction profile kernels for predicting drug–target interaction. Bioinformatics 27 (21), 3036–3043. 10.1093/bioinformatics/btr500 21893517

[B46] Vilar S. Hripcsak G. (2016). The role of drug profiles as similarity metrics: applications to repurposing, adverse effects detection and drug–drug interactions. Briefings In Bioinf. 18 (4), 670–681. 10.1093/bib/bbw048 PMC6078166 27273288

[B47] Waris M. Ahmad K. Kabir M. Hayat M. (2016). Identification of DNA binding proteins using evolutionary profiles position specific scoring matrix. Neurocomputing 199, 154–162. 10.1016/j.neucom.2016.03.025

[B48] Wishart D. S. Knox C. Guo A. C. Cheng D. Shrivastava S. Tzur D. (2007). DrugBank: a knowledgebase for drugs, drug actions and drug targets. Nucleic Acids Res. 36 (suppl_1), D901–D906. 10.1093/nar/gkm958 18048412 PMC2238889

[B49] Xia Z. Wu L.-Y. Zhou X. Wong S. T. (2010). Semi-supervised drug-protein interaction prediction from heterogeneous biological spaces. BMC systems biology. 4 (2), S6. 10.1186/1752-0509-4-S2-S6 PMC2982693 20840733

[B50] Yamanishi Y. Araki M. Gutteridge A. Honda W. Kanehisa M. (2008). Prediction of drug–target interaction networks from the integration of chemical and genomic spaces. Bioinformatics 24 (13), i232–i240. 10.1093/bioinformatics/btn162 18586719 PMC2718640

[B51] Zheng X. Ding H. Mamitsuka H. Zhu S. (2013). Collaborative matrix factorization with multiple similarities for predicting drug-target interactions In *Proceedings of the 19th ACM SIGKDD international conference on Knowledge discovery and data mining* p. 1025–1033. 10.1145/2487575.2487670

