# Supplementary material for: A Novel Approach for Drug-Target Interactions Prediction Based on Multimodal Deep Autoencoder
Source: Front Pharmacol. 2020 Jan 28;10:1592. doi: 10.3389/fphar.2019.01592 (PMC6997437; doi:10.3389/fphar.2019.01592)
Supplement: Supplementary file 1 [file DataSheet_1.docx]

Supplementary Material

Table S1. The hyperparameters of MDADTI model for five datasets under CVS1 setting

|  | NR | GPCR | IC | E | DrugBank_FDA |
| --- | --- | --- | --- | --- | --- |
| Learning rate | 0.0001 | 0.001 | 0.001 | 0.001 | 0.001 |
| Epoch | 100 | 200 | 200 | 200 | 300 |
| Batchsize | 64 | 64 | 256 | 256 | 256 |
| Momentum | 0.90 | 0.90 | 0.90 | 0.90 | 0.90 |

Table S2. The hyperparameters of MDADTI model for four datasets under CVS2 setting

|  | NR | GPCR | IC | E | DrugBank_FDA |
| --- | --- | --- | --- | --- | --- |
| Learning rate | 0.0001 | 0.001 | 0.001 | 0.001 | 0.001 |
| Epoch | 100 | 100 | 100 | 100 | 100 |
| Batchsize | 64 | 64 | 64 | 256 | 256 |
| Momentum | 0.90 | 0.90 | 0.90 | 0.90 | 0.90 |

Table S3. The hyperparameters of MDADTI model for four datasets under CVS3 setting

|  | NR | GPCR | IC | E | DrugBank_FDA |
| --- | --- | --- | --- | --- | --- |
| Learning rate | 0.0001 | 0.001 | 0.001 | 0.001 | 0.001 |
| Epoch | 100 | 100 | 100 | 100 | 100 |
| Batchsize | 64 | 64 | 64 | 256 | 256 |
| Momentum | 0.90 | 0.90 | 0.90 | 0.90 | 0.90 |

Table S4 The p-values of paired t-test between our method MDADTI and DDR, KronRLS-MKL, NRLMF,

BLM-NII on five datasets

(a) CVS1 setting

|  |  | DDR | KronRLS-MKL | NRLMF | BLM-NII |
| --- | --- | --- | --- | --- | --- |
| NR | AUC | **3.998**$\boldsymbol{\times}$**10^-3^** | **5.165**$\boldsymbol{\times}$**10^-4^** | 0.067 | **5.574**$\boldsymbol{\times}$**10^-3^** |
|  | AUPR | **5.286**$\boldsymbol{\times}$**10^-4^** | **5.356**$\boldsymbol{\times}$**10^-4^** | **5.496**$\boldsymbol{\times}$**10^-4^** | **5.721**$\boldsymbol{\times}$**10^-4^** |
| GPCR | AUC | **1.499**$\boldsymbol{\times}$**10^-3^** | **5.672×10^-4^** | **5.342**$\boldsymbol{\times}$**10^-4^** | **5.248**$\boldsymbol{\times}$**10^-4^** |
|  | AUPR | **5.793**$\boldsymbol{\times}$**10^-4^** | **5.689**$\boldsymbol{\times}$**10^-4^** | **5.751**$\boldsymbol{\times}$**10^-4^** | **5.841**$\boldsymbol{\times}$**10^-4^** |
| IC | AUC | 0.072 | **0.011** | **5.683**$\boldsymbol{\times}$**10^-4^** | **8.496**$\boldsymbol{\times}$**10^-3^** |
|  | AUPR | **5.835**$\boldsymbol{\times}$**10^-4^** | **5.643**$\boldsymbol{\times}$**10^-4^** | **5.479**$\boldsymbol{\times}$**10^-4^** | **5.679**$\boldsymbol{\times}$**10^-4^** |
| E | AUC | 0.391 | 0.465 | 0.445 | 0.360 |
|  | AUPR | **5.467**$\boldsymbol{\times}$**10^-4^** | **5.568**$\boldsymbol{\times}$**10^-4^** | **5.256**$\boldsymbol{\times}$**10^-4^** | **5.233**$\boldsymbol{\times}$**10^-4^** |
| DrugBank_FDA | AUC | - | - | **5.124**$\boldsymbol{\times}$**10^-4^** | **5.486**$\boldsymbol{\times}$**10^-4^** |
|  | AUPR | - | - | **5.334**$\boldsymbol{\times}$**10^-4^** | **5.317**$\boldsymbol{\times}$**10^-4^** |

(b) CVS2 setting

|  |  | DDR | KronRLS-MKL | NRLMF | BLM-NII |
| --- | --- | --- | --- | --- | --- |
| NR | AUC | **5.671**$\boldsymbol{\times}$**10^-4^** | 0.068 | **0.005** | **0.009** |
|  | AUPR | **5.497**$\boldsymbol{\times}$**10^-3^** | **5.320**$\boldsymbol{\times}$**10^-4^** | **5.112**$\boldsymbol{\times}$**10^-4^** | **5.232**$\boldsymbol{\times}$**10^-4^** |
| GP  CR | AUC | **1.499**$\boldsymbol{\times}$**10^-3^** | **5.682×10^-4^** | 0.964 | 0.740 |
|  | AUPR | **5.124**$\boldsymbol{\times}$**10^-4^** | **5.341**$\boldsymbol{\times}$**10^-4^** | **5.043**$\boldsymbol{\times}$**10^-4^** | **5.793**$\boldsymbol{\times}$**10^-4^** |
| IC | AUC | **5.085**$\boldsymbol{\times}$**10^-4^** | 0.616 | **0.014** | **5.032**$\boldsymbol{\times}$**10^-4^** |
|  | AUPR | 0.244 | **5.439**$\boldsymbol{\times}$**10^-4^** | **5.648**$\boldsymbol{\times}$**10^-4^** | **5.110**$\boldsymbol{\times}$**10^-4^** |
| E | AUC | **5.314**$\boldsymbol{\times}$**10^-4^** | **5.519**$\boldsymbol{\times}$**10^-4^** | **0.027** | **0.016** |
|  | AUPR | 0.116 | **5.317**$\boldsymbol{\times}$**10^-4^** | **5.421**$\boldsymbol{\times}$**10^-4^** | **5.938**$\boldsymbol{\times}$**10^-3^** |
| DrugBank_FDA | AUC | - | - | **5.029**$\boldsymbol{\times}$**10^-4^** | **5.137**$\boldsymbol{\times}$**10^-4^** |
|  | AUPR | - | - | **5.301**$\boldsymbol{\times}$**10^-4^** | **5.524**$\boldsymbol{\times}$**10^-4^** |

(c) CVS3 setting

|  |  | DDR | KronRLS-MKL | NRLMF | BLM-NII |
| --- | --- | --- | --- | --- | --- |
| NR | AUC | **5.101**$\boldsymbol{\times}$**10^-4^** | **5.274**$\boldsymbol{\times}$**10^-4^** | **5.031**$\boldsymbol{\times}$**10^-4^** | **5.092**$\boldsymbol{\times}$**10^-4^** |
|  | AUPR | **0.0025** | 0.673 | 0.830 | 0.553 |
| GP  CR | AUC | **5.367**$\boldsymbol{\times}$**10^-4^** | **5.281×10^-4^** | **5.458**$\boldsymbol{\times}$**10^-4^** | 0.57 |
|  | AUPR | **5.901**$\boldsymbol{\times}$**10^-4^** | **5.833**$\boldsymbol{\times}$**10^-4^** | **5.541**$\boldsymbol{\times}$**10^-4^** | **5.001**$\boldsymbol{\times}$**10^-4^** |
| IC | AUC | **5.743**$\boldsymbol{\times}$**10^-4^** | **5.433**$\boldsymbol{\times}$**10^-4^** | **0.008** | **5.689**$\boldsymbol{\times}$**10^-4^** |
|  | AUPR | **0.001** | **5.413**$\boldsymbol{\times}$**10^-4^** | **5.624**$\boldsymbol{\times}$**10^-4^** | **5.112**$\boldsymbol{\times}$**10^-4^** |
| E | AUC | **5.106**$\boldsymbol{\times}$**10^-4^** | **5.142**$\boldsymbol{\times}$**10^-4^** | **5.343**$\boldsymbol{\times}$**10^-4^** | **5.142**$\boldsymbol{\times}$**10^-4^** |
|  | AUPR | **5.217**$\boldsymbol{\times}$**10^-4^** | **5.628**$\boldsymbol{\times}$**10^-4^** | **5.441**$\boldsymbol{\times}$**10^-4^** | **5.399**$\boldsymbol{\times}$**10-^4^** |
| DrugBank_FDA | AUC | - | - | **5.639**$\boldsymbol{\times}$**10^-4^** | **5.017**$\boldsymbol{\times}$**10^-4^** |
|  | AUPR | - | - | **5.172**$\boldsymbol{\times}$**10^-4^** | **5.485**$\boldsymbol{\times}$**10^-4^** |

*P-value in bold is less than 0.05, which indicates that the differences between our method and the baseline method are statistically significant at the significance level of 0.05.

Table S5. The fractions of validated DTIs of MDADTI, NRLMF, BLM-NII among the predicted Top N

(N = 10, 30, 50) DTIs in four datasets

| Method | NR | | | GPCR | | | IC | | | E | | |
| --- | --- | --- | --- | --- | --- | --- | --- | --- | --- | --- | --- | --- |
|  | Top  10 | Top  30 | Top  50 | Top  10 | Top  30 | Top  50 | Top  10 | Top  30 | Top  50 | Top  10 | Top  30 | Top  50 |
| BLM-NII | 30% | 27% | 16% | 70% | 60% | 58% | 30% | 30% | 34% | 70% | 60% | 46% |
| NRLMF | 50% | 43% | 28% | 60% | 67% | 60% | 50% | 33% | 34% | 90% | 60% | 44% |
| MDADTI | 50% | 43.3% | 28% | 80% | 66.67% | 60% | 80% | 50% | 40% | 100% | 73.33% | 52% |

*The fractions of validated DTIs of NRLMF, BLM-NII among the predicted top N (N = 10, 30, 50) DTIs in four datasets are provided by (Liu et al., 2016).

Table S6 The comparison of AUC and AUPR between MDADTI with transfer learning and MDADTI without transfer learning on NR dataset

|  |  | AUC | AUPR |
| --- | --- | --- | --- |
| CVS1 | With transfer learning | 0.966 | 0.959 |
|  | Without transfer learning | 0.766 | 0.750 |
| CVS2 | With transfer learning | 0.80 | 0.78 |
|  | Without transfer learning | 0.65 | 0.63 |
| CVS3 | With transfer learning | 0.60 | 0.44 |
|  | Without transfer learning | 0.58 | 0.44 |

*The hyperparameters of MDADTI without transfer learning are the same with that of MDADTI with transfer learning which are listed in Table S1, Table S2 and Table S3.The MDA extracting the drug features is configured as $\left[ n\boldsymbol{*}n_{d},25,n\boldsymbol{*}n_{d} \right]$, and the MDA extracting target features is configured as $[n\boldsymbol{*}n_{t},10,n\boldsymbol{*}n_{t}]$ when we applied MDADTI without transfer learning to predict DTIs in NR dataset.

Table S7 The comparison of AUC and AUPR between MDADTI with SMOTE and MDADTI without SMOTE method on NR, GPCR, IC, E and DrugBank_FDA dataset

(a) The comparison of AUC

|  |  | NR | GPCR | IC | E | Drugbank_FDA |
| --- | --- | --- | --- | --- | --- | --- |
| CVS1 | With SMOTE | 0.966 | 0.980 | 0.991 | 0.983 | 0.963 |
|  | Without SMOTE | 0.664 | 0.813 | 0.753 | 0.887 | 0.769 |
| CVS2 | With SMOTE | 0.80 | 0.87 | 0.76 | 0.79 | 0.94 |
|  | Without SMOTE | 0.50 | 0.73 | 0.64 | 0.63 | 0.63 |
| CVS3 | With SMOTE | 0.60 | 0.86 | 0.94 | 0.94 | 0.91 |
|  | Without SMOTE | 0.51 | 0.71 | 0.73 | 0.70 | 0.62 |

(b) The comparison of AUPR

|  |  | NR | GPCR | IC | E | Drugbank_FDA |
| --- | --- | --- | --- | --- | --- | --- |
| CVS1 | With SMOTE | 0.959 | 0.978 | 0.987 | 0.980 | 0.959 |
|  | Without SMOTE | 0.140 | 0.189 | 0.145 | 0.428 | 0.056 |
| CVS2 | With SMOTE | 0.78 | 0.86 | 0.73 | 0.81 | 0.95 |
|  | Without SMOTE | 0.08 | 0.08 | 0.06 | 0.02 | 0.01 |
| CVS3 | With SMOTE | 0.44 | 0.87 | 0.96 | 0.96 | 0.92 |
|  | Without SMOTE | 0.09 | 0.08 | 0.11 | 0.03 | 0.01 |


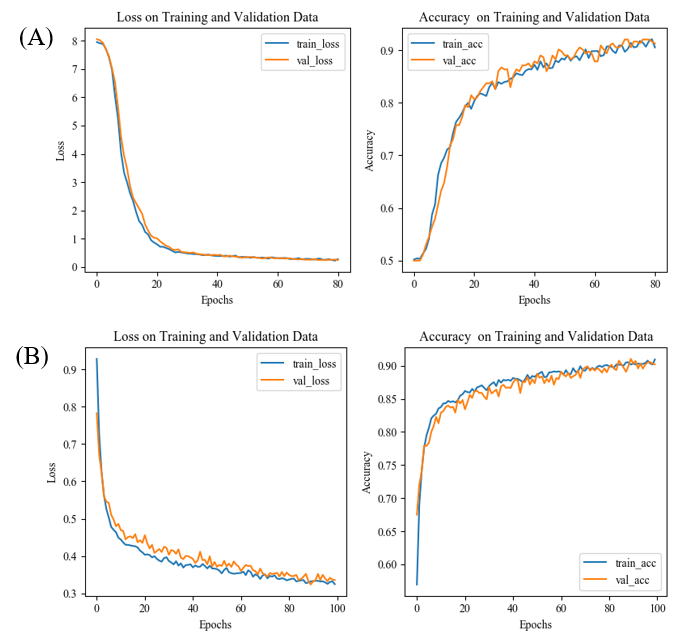


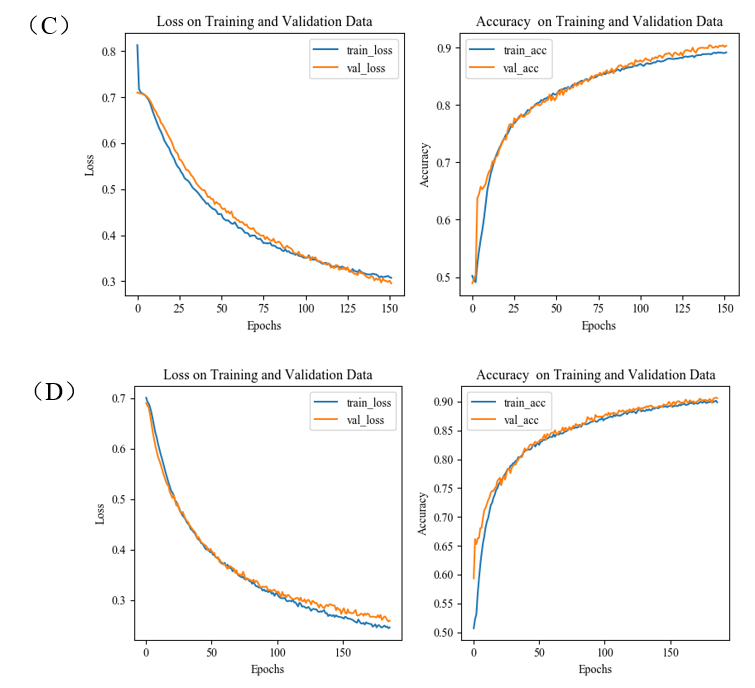


Figure S1. The train/valid accuracy-epoch and loss-epoch curves of first repeat of 10-fold cross validation while selecting fold1 as test set and the remaining as train set for NR, GPCR, IC and E datasets. The left is the loss-epoch curve, and the right is accuracy-epoch curve.(A) The train/valid accuracy-epoch and loss-epoch curves for NR dataset;(B) The train/valid accuracy-epoch and loss-epoch curves for GPCR dataset; (C) The train/valid accuracy-epoch and loss-epoch curves for IC dataset; (D) The train/valid accuracy-epoch and loss-epoch curves for E dataset. These four figures show that the change law of accuracy and loss of our model while validating are consistent with that while training, which demonstrates that overfitting has been effectively processed for each dataset.


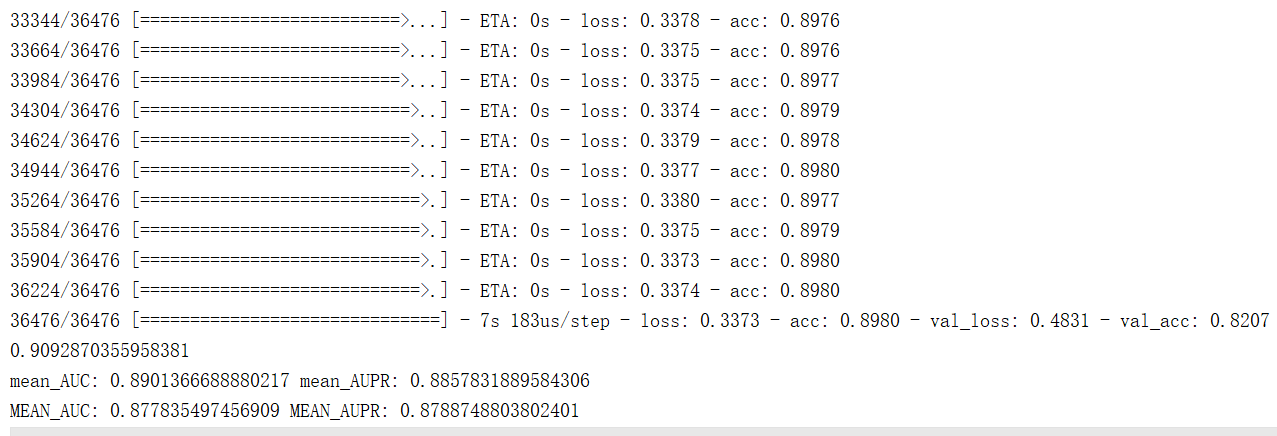


Figure S2. The screenshot of MDADTI when it predicts DTIs in GPCR dataset under CVS2 setting


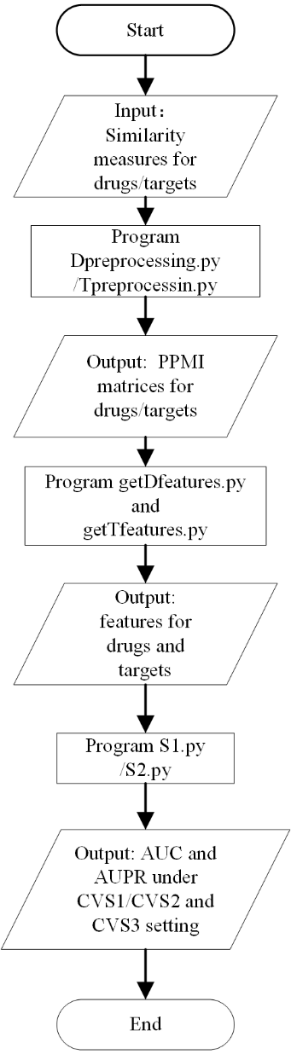


Figure S3. The program flow chart of the code. **Step 1:** Run “Python Dpreprocessing.py ic” command directly, and perform random walk with restart method and calculate positive pointwise mutual information; **Step 2:** Execute the command “Python getDfeatures.py deepNF_params.txt” to fuse multiple positive pointwise mutual information (PPMI) matrices with multimodal deep autoencoder; **Step 3:** Run S1.py/S2.py file directly to concatenate the drug and target features ​​together and then pass them into the DNN.
